# Teflon promotes chromosomal recruitment of homolog conjunction proteins during *Drosophila* male meiosis

**DOI:** 10.1371/journal.pgen.1010469

**Published:** 2022-10-17

**Authors:** Zeynep Kabakci, Hiro Yamada, Luisa Vernizzi, Samir Gupta, Joe Weber, Michael Shoujie Sun, Christian F. Lehner

**Affiliations:** Department of Molecular Life Science (DMLS), University of Zurich, Zurich, Switzerland; Stowers Institute for Medical Research, UNITED STATES

## Abstract

Meiosis in males of higher dipterans is achiasmate. In their spermatocytes, pairing of homologs into bivalent chromosomes does not include synaptonemal complex and crossover formation. While crossovers preserve homolog conjunction until anaphase I during canonical meiosis, an alternative system is used in dipteran males. Mutant screening in *Drosophila melanogaster* has identified *teflon* (*tef*) as being required specifically for alternative homolog conjunction (AHC) of autosomal bivalents. The additional known AHC genes, *snm*, *uno* and *mnm*, are needed for the conjunction of autosomal homologs and of sex chromosomes. Here, we have analyzed the pattern of TEF protein expression. TEF is present in early spermatocytes but cannot be detected on bivalents at the onset of the first meiotic division, in contrast to SNM, UNO and MNM (SUM). TEF binds to polytene chromosomes in larval salivary glands, recruits MNM by direct interaction and thereby, indirectly, also SNM and UNO. However, chromosomal SUM association is not entirely dependent on TEF, and residual autosome conjunction occurs in *tef* null mutant spermatocytes. The higher *tef* requirement for autosomal conjunction is likely linked to the quantitative difference in the amount of SUM protein that provides conjunction of autosomes and sex chromosomes, respectively. During normal meiosis, SUM proteins are far more abundant on sex chromosomes compared to autosomes. Beyond promoting SUM recruitment, TEF has a stabilizing effect on SUM proteins. Increased SUM causes excess conjunction and consequential chromosome missegregation during meiosis I after co-overexpression. Similarly, expression of SUM without TEF, and even more potently with TEF, interferes with chromosome segregation during anaphase of mitotic divisions in somatic cells, suggesting that the known AHC proteins are sufficient for establishment of ectopic chromosome conjunction. Overall, our findings suggest that TEF promotes alternative homolog conjunction during male meiosis without being part of the final physical linkage between chromosomes.

## Author summary

Sexual reproduction depends on meiosis, a special cell division that generates haploid cells. Haploid cells have only one set of chromosomes in contrast to the diploid precursor cell, which has two sets. Haploid cells can differentiate into gametes. Fusion of two gametes during fertilization recreates the diploid state. Meiosis is distinct in males and females to produce two distinct types of compatible gametes, sperm and egg. In the fly *Drosophila melanogaster*, sex-specific differences are particularly pronounced. While pairing of homologous chromosomes into bivalents early in meiosis proceeds in a canonical manner in females, males use an alternative system. This system maintains homolog pairing, replacing crossovers that result from homologous recombination during canonical meiosis. Four genes (*snm*, *uno*, *mnm* and *tef*) are known to be required specifically for alternative homolog conjunction in males. Here, we demonstrate that the TEF protein binds directly to MNM. Thereby, TEF promotes the recruitment of MNM and consequentially SNM and UNO to chromosomes. However, while SNM, UNO and MNM remain on bivalent chromosomes until they are separated apart during the first meiotic division, TEF disappears prematurely, suggesting that it is not part of the final physical linkage between homologous chromosomes.

## Introduction

Meiosis is a key innovation that evolved before the eukaryotic radiation into the extant domain. The canonical program of this conserved process relies on meiotic recombination (MR). MR contributes to the initial pairing of homologous chromosomes and generates crossovers that maintain homologs linked as bivalent chromosomes until the onset of anaphase during the first meiotic division (M I). MR proceeds usually in concert with synapsis, which achieves close homolog pairing all along the chromosomes via formation of the synaptonemal complex (SC). In spite of the eminent significance of MR, diverse meiosis variants have evolved that do not rely on MR [1]. A most thoroughly studied example of such an achiasmate meiosis occurs in *Drosophila melanogaster*. While meiosis is largely canonical in *D*. *melanogaster* females and includes MR, it is achiasmate in the heterogametic males. This sex-specific difference in meiosis is characteristic among higher dipterans. Its evolution is poorly understood, but may be linked to the suppression of recombination between sex chromosomes [2].

In *D*. *melanogaster* spermatocytes, not only MR but also SC formation does not occur. Nevertheless, soon after the last spermatogonial mitosis, homologous chromosomes are paired all along their length, according to analyses with a lacO/lacI-GFP system and FISH [3,4]. It remains to be clarified whether the pairing of homologous chromosomes in early spermatocytes during the S1 stage is driven by the same mechanisms that are responsible for the pervasive somatic homolog pairing in *D*. *melanogaster* [5]. Importantly, the extensive pairing of homologs in spermatocytes lasts only a few hours. During the S2b/S3 stages, homolog pairing was no longer detectable at any of the analyzed 14 distinct locations with euchromatic lacO array insertions [3]. Moreover, even sister chromatid cohesion appeared to be lost except at centromeres [3].

The drastic loss of homolog pairing and sister cohesion in mid-stage spermatocytes starts concomitantly with the process of territory formation, which separates three major chromosome territories apart within the interphase nucleus. One of the major territories contains the chromosome (chr) 2 bivalent, another the chr3 bivalent and the third the chrXY bivalent. The additional bivalent of chr4, a small dot chromosome, is often associated with the chrXY territory. Territory formation breaks up all non-homologous associations between the large chromosomes. Such non-homologous associations are extensive in S1 spermatocytes. They arise from a coalescence of large blocks of pericentromeric heterochromatin into a chromocenter. Similarly, centromeres are clustered initially. Disrupting these non-homologous associations during territory formation at the S2b stage depends on condensin II activity and additional unidentified forces [6,7]. Failure of territory formation leads to persistence of non-homologous associations until prometaphase I and consequential chromosome segregation errors [6,7].

The mechanisms that break up non-homologous chromosome associations during territory formation disrupt also homolog pairing and sister chromatid cohesion, presumably because of inevitable side effects. However, normally, homolog separation does not proceed to completion already during spermatocyte maturation. Complete premature homolog separation is prevented by residual homolog conjunction maintained by a dedicated special system that serves as an alternative to canonical homolog linkage by crossovers. Large-scale mutant screening has led to the identification of three genes (*tef*, *mnm*, and *snm*) that are specifically required for this alternative homolog conjunction (AHC) [8–10]. A proteomic approach has recently uncovered an additional AHC gene (*uno*) [11]. Loss-of-function mutations in these four genes result in chromosome missegregation during M I, but exclusively in males. In *mnm*, *snm* and *uno* mutant males, both sex chromosomes and autosomes are distributed randomly during M I [10,11]. In contrast, only autosomes are missegregated in *tef* mutant males during M I [8,9].

The TEF protein includes three C2H2-type zinc fingers and is therefore predicted to bind to DNA [9]. The SNM protein is a distant relative of the stromalins (SCC3/SA/STAG protein family) [10]. Stromalins are subunits of cohesins, complexes of crucial importance for chromosome organization during interphase and M phase in somatic and meiotic cells. However, SNM is not co-localized with core components of cohesin, indicating that it does not function as a cohesin subunit [10]. MNM is encoded by one of many differentially spliced mRNAs transcribed from the highly complex *mod(mdg4)* locus [10,12]. MNM has an N-terminal BTB/POZ motif that is shared among almost all of the more than 30 distinct protein products expressed from the *mod(mdg4)* locus [10,12,13]. In addition, MNM has a unique C-terminal zinc finger motif of the FLYWCH type. These N- and C-terminal motifs of MNM are predicted to mediate protein-protein interactions [14]. UNO does not have obvious similarities to functionally characterized proteins [11].

MNM, SNM and UNO accumulate in early spermatocytes, eventually co-localizing during spermatocyte maturation in multiple subnucleolar foci [10,11]. At the start of M I, these foci coalesce into a single prominent spot on the chrXY bivalent [10,11]. In *D*. *melanogaster*, chrX and chrY are strongly heteromorphic, lacking extended euchromatic homology that could mediate specific pairing. However, both sex chromosomes harbor rDNA gene clusters in the centromere-proximal heterochromatin and these rDNA clusters function as pairing centers during male M I [15,16]. The prominent dot formed by MNM, SNM and UNO on the chrXY bivalent at the start of M I is localized on the paired rDNA loci of chrX and chrY [10,16]. Apart from the prominent dot on the chrXY pairing center, autosomal bivalents, which rely on euchromatic homology for pairing [17,18] display far weaker dot signals of co-localized MNM, SNM and UNO [10,11]. These autosomal dot signals were shown to be at least partially dependent on *tef* function [10,11]. Strikingly, MNM, SNM and UNO disappear rapidly from all the bivalents within minutes during the onset of anaphase I [10,11,19]. Separase, an endoprotease known to eliminate chromosomal cohesin at the metaphase to anaphase transition during mitotic and meiotic divisions, is required for the rapid disappearance of MNM, SNM and UNO from M I bivalents [19]. UNO includes a separase cleavage site [11]. Mutations that abolish this cleavage site prevent the rapid disappearance of MNM, SNM and UNO from M I bivalents and preclude homolog separation [11].

The findings summarized above strongly support the notion that SNM, MNM and UNO function as proteinaceous glue that conjoins chromosomes into bivalents. However, it remains to be clarified how these proteins are recruited to chromosomes. SNM, MNM and UNO do not include known *bona fide* DNA-binding domains. They might therefore be recruited by other chromatin proteins. The zinc finger protein TEF is clearly an attractive candidate factor for chromosomal recruitment of the other AHC proteins. TEF’s pattern of expression and its subcellular localization during spermatogenesis have not yet been characterized. Here, we close this gap in understanding. Using transgenes encoding tagged functional versions of TEF, we observed that it is only transiently detectable in early spermatocytes. In contrast to the other known AHC proteins (MNM, SNM and UNO), TEF cannot be detected on bivalents at the start of M I, indicating that it is unlikely a stoichiometric component of the homolog-conjoining glue. However, we provide evidence that TEF can recruit MNM to chromosomes by direct protein-protein interaction. Indirectly, TEF can also recruit SNM-UNO, as they bind to MNM. Moreover, presumably by promoting AHC protein interactions, TEF stabilizes these proteins and controls their levels. AHC protein levels need to be controlled, as suggested by the consequences of simultaneous overexpression of all four AHC proteins in spermatocytes, which resulted in ectopic chromosome conjunction, failure of territory formation and segregation errors during M I. Ectopic expression of the four AHC proteins in somatic cells induced aberrant chromosome conjunction during mitosis, suggesting that AHC might not depend on additional spermatocyte-specific proteins beyond those already known.

## Results

### Residual autosomal homolog conjunction in *tef* mutants

**Fig 1:**
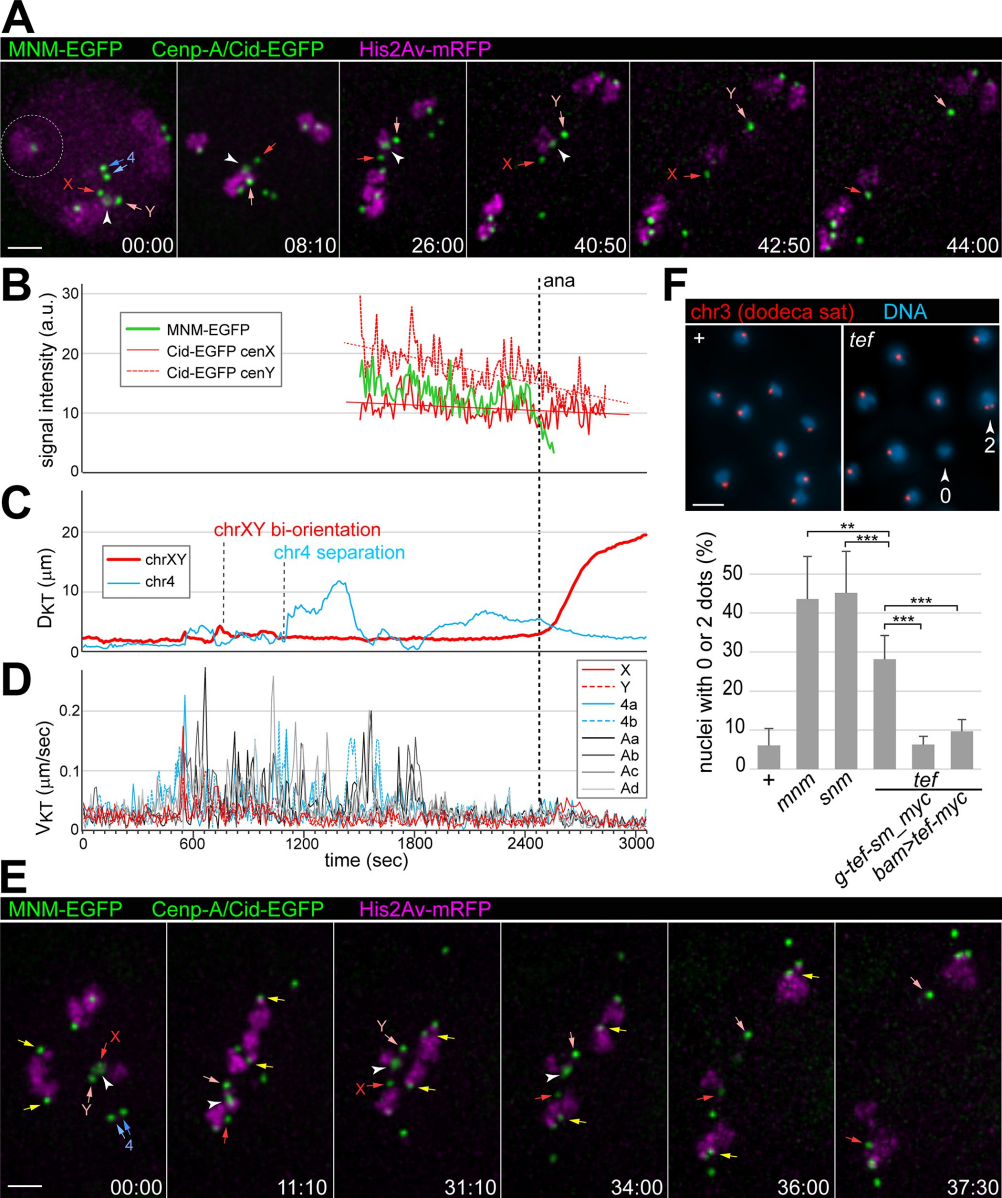
Autosome segregation in M I is less defective in *tef* compared to *mnm* and *snm* mutants. **(A-E)** Time-lapse imaging of progression through M I in *tef* mutants expressing His2Av-mRFP, Cenp-A/Cid-EGFP and MNM-EGFP. Autosome conjunction defects of varying severity are illustrated with a severely **(A-D)** and a moderately affected cell **(E)**. **(A)** Stills from selected time points (min:sec) revealing an isolated univalent (dashed circle) at the onset of NEBD I (00:00), as well as three bivalents formed by a large autosome, chr4 and chrXY, respectively. The centromeric Cid-EGFP signals (arrows) associated with the bivalents of chr4 (blue) and the sex chromosomes (red) are indicated, as well as the MNM-EGFP signal (white arrowhead), marking the sex chromosome pairing region. While the chr4 bivalents was separated prematurely (see C), the chrXY bivalent remained in a stable equatorial position after bi-orientation (see 26:00 and 40:50) until regular separation during anaphase onset, accompanied by MNM-EGFP disappearance (see 42:50 and 44:00). **(B)** EGFP dot signal intensities associated with the KTs of chrX and chrY, as well as with their pairing region were quantified to monitor the activation of the Anaphase-Promoting Complex/Cyclosome (APC/C). Single exponential curves fitted to the Cid-EGFP signals revealed their stability apart from photobleaching, while MNM-EGFP disappeared rapidly during exit from M I. Onset of anaphase I as revealed by KT movements is indicated (dashed line). **(C)** Distances (D_KT_) between the two KTs of the sex chromosome bivalent (red) and of the chr4 bivalent (blue), respectively, were measured to monitor bivalent separation. **(D)** KT velocities (V_KT_) were measured after tracking all eight KTs during M I. Starting about 420 seconds after NEBD I onset, rapid KT jumps resulting from initial interactions with spindle MTs were observed. In contrast to the rapid stabilization of the sex chromosome bivalent by bi-orientation (around 690 sec), univalents displayed KT jumps back and forth between the spindle poles over an extended period before delayed stabilization (at around 1900 sec). **(E)** A *tef* mutant cell with apparently normal bivalents just after NEBD I (00:00). While two of the three autosomal bivalents were separated prematurely, the third large autosomal bivalent (KTs indicated by yellow arrows) was bi-oriented normally in parallel with the chrXY bivalent (see 31:10) until regular separation and segregation during anaphase I (see 34:00, 36:00 and 37:30). **(F)** FISH with a fluorescent probe for the dodeca satellite on chr3 was used to determine the extent of meiotic chromosome missegregation. After normal segregation, as in *w* (+), a single FISH signal is present in the large majority of the early spermatid nuclei. In contrast, missegregation during M I, as in *tef* mutants (*tef*), results in spermatid nuclei with either zero or two FISH signals, as indicated. Bars reveal the average percentage of nuclei with either zero or two dodeca FISH signals after analysis of multiple spermatid cysts for each of the indicated genotypes. Standard deviations are shown as well. n = 18 (+), 9 (*mnm*), 9 (*snm*), 10 (*tef*), 5 (*tef*, *g-tef-sm_myc*), and 5 (*tef*, *bam>tef-myc*). Chr3 missegregation is significantly lower in *tef* mutants compared to *mnm* or *snm* mutants. Moreover, expression of *g-tef-sm_myc* or *bam>tef-myc* significantly reduced chr3 missegregation in *tef* mutants to a level that was not significantly different from control (+). ** p < .01, *** p < .001. Scale bars = 3 μm (A,E) and 5 μm (F).

By time-lapse imaging of meiosis with high temporal and spatial resolution in *Drosophila* spermatocytes [20], we characterized the *tef* mutant phenotype (Fig 1A–1E and S1 Movie). Previous cytological analyses were achieved with fixed samples [8–10]. For live imaging, we used *tef* mutants (*tef*^z2-3455^/*tef*^z2-4169^) expressing fluorescent fusion proteins marking chromosomes (His2Av-mRFP) and centromeres (Cenp-A/Cid-EGFP). Chromosome-specific features revealed by these markers permit an unequivocal identification of chrXY and chr4 bivalents in normal spermatocytes [20,21]. The sex chromosome bivalent stands out because of its association with the strongest centromeric Cid-EGFP dot, resulting from the two-fold higher levels of Cenp-A/Cid in the chrY centromere compared to the other centromeres. The small chr4 has a very low level of associated His2Av-mRFP. The additional two bivalents in normal spermatocytes are those formed by the large autosomes, chr2 and chr3. Beyond Cid-EGFP and His2Av-mRFP, the *tef* mutants that were used for time-lapse imaging also expressed an *mnm-EGFP* transgene under the control of *Hsp70 cis*-regulatory sequences [10]. MNM-EGFP resulting from basal expression (without heat shocks) marks the sex chromosome pairing region very prominently, while the far weaker signals on autosomal bivalents were not evident with the chosen imaging and display settings. The MNM-EGFP dot on the chrXY pairing region could be discriminated readily from centromeric Cenp-A/Cid-EGFP signals. The MNM-EGFP dot was less focused and disappeared rapidly during anaphase I (Fig 1A and 1B and 1E and S1 Movie). As expected [8], our time-lapse imaging revealed that the sex chromosome bivalent behaved normally during M I in *tef* mutants (Fig 1A–1E and S1 Movie) (n = 7 spermatocytes from three different cysts). As in controls that have been previously characterized in detail using identical methods [20], the chrXY bivalents attained bi-orientation within less than 15 minutes after nuclear envelope breakdown (NEBD) I. In the spermatocyte shown in Fig 1A and S1 Movie, kinetochore (KT) jumps, indicating interactions between bivalents and spindle microtubules (MTs), started about seven minutes after the onset of NEBD I. Five minutes later, the chrXY bivalent had reached bi-orientation and remained at a relatively stable equatorial position, as also indicated by the reduced velocity of the associated chrX and chrY KTs (V_KT_) (Fig 1D) and by the increase in their spatial separation (D_kt_) by about 15% (Fig 1C). After about 30 minutes of stable bi-orientation, the chrXY bivalent was split concomitant with MNM-EGFP disappearance, and the sex chromosomes were pulled apart to opposite spindle poles (Fig 1A–1D and S1 Movie). In contrast to the entirely normal bi-orientation of the sex chromosome bivalent, autosomal bivalents were prone to premature separation in *tef* mutants. While some autosomes were present as widely separated univalents already before NEBD I, others were still conjoined into apparently normal bivalents at the start of M I. In the spermatocyte shown in Fig 1A–1D and S1 Movie, chr4 and one large autosome were still present in form of bivalents at the time of NEBD I, while the second large autosome was disjoined, with one univalent forming a separate distinct chromosome territory and the other univalent being localized within a territory that also contained the bivalents of chrXY and chr4. Some of the autosomal bivalents that were still intact at NEBD I in *tef* mutants, were ripped apart before the metaphase-to-anaphase I transition (scored based on the sex chromosome behavior). This premature bivalent separation was accompanied by rapid KT jumps, indicating the action of forces mediated by spindle MTs. In case of the spermatocyte shown in Fig 1A–1D and S1 Movie, the premature separation of the chr4 bivalent was definitive about 18 minutes after NEBD I, i.e. long before anaphase I onset (see Fig 1C). Univalents usually continued to jump back and forth between spindle poles (Fig 1D and S1 Movie), as previously described in *mnm* and *snm* mutants [20]. Without physical linkage between homologs, KT-MT attachments cannot be stabilized by mechanical tension, as after bi-orientation of normal bivalents. The prolonged presence of unattached KTs in *tef* mutants presumably results in activation of the spindle assembly checkpoint, explaining the slight delay of the metaphase-to-anaphase I transition by 5–10 min [20]. Nevertheless, univalents eventually adopted a relatively stable position usually off the equatorial plane and near to a spindle pole (Fig 1A and 1D and S1 Movie).

Interestingly, not all autosomal bivalents were prematurely separated in *tef* mutant spermatocytes. As illustrated in Fig 1E, some were bi-oriented rather normally, remaining in a stable equatorial position before separating slightly ahead of the sex chromosomes, as during normal M I [11,20]. An autosomal bivalent with apparently normal behavior was detected in four out of the seven *tef* mutant spermatocytes, in which the KTs were tracked at high spatial and temporal resolution.

The presence of normal autosomal bivalents in *tef* mutants during M I suggested that autosomal homolog conjunction might be less defective in these mutants compared to *mnm*, *snm* and *uno* mutants, which were analyzed earlier by analogous time-lapse imaging [11,19,20]. To confirm the apparent difference, we quantified chr3 missegregation during meiosis in *tef*, *mnm* and *snm* mutants with fluorescence *in situ* hybridization (FISH) and a probe targeting the chr3-specific dodeca satellite [22]. After hybridization to testis squash preparations, the number of nuclei in early spermatid cysts with either zero, one or two probe dots was determined (Fig 1F and S4 Table). In control testis, as expected, the overwhelming majority of spermatid nuclei (94%) displayed one dodeca FISH signal (Fig 1F). In *mnm* and *snm* mutants, the fraction of spermatid nuclei with one signal was severely reduced to 57% and 54%, respectively (Fig 1F). Importantly, in *tef* mutants, the fraction of nuclei with one signal (72%) was significantly higher than in *mnm* and *snm* mutants (Fig 1F) (see Materials and Methods for additional comments on the dodeca FISH assay). Overall, we conclude that the autosomal conjunction defect is less pronounced in *tef* mutants compared to *mnm* and *snm* mutants. The *tef* alleles used in our analyses harbor early premature stop codons and were reported to behave as null alleles [9]. The presence of residual autosome conjunction in complete absence of *tef* function argues against models where homolog linkage in autosomal bivalents is maintained at the start of M I in a manner that is strictly dependent on the physical presence and interaction of TEF with one or several of the other AHC proteins (SNM, MNM and UNO).

### TEF-EGFP sufficient for rescue is detectable in early but not in late spermatocytes (in contrast to MNM-, SNM- and UNO-EGFP)

The subcellular localization of TEF during spermatogenesis might provide important insights concerning the role of this protein in AHC. As the ‘spaghetti monster’ fluorescent proteins (smFPs) were recently developed and reported to allow successful immunolabeling of weakly expressed proteins [23], we generated transgenic lines (*g*-*tef-sm_myc*) expressing TEF fused to such a tag. The C-terminal sm_myc tag consists of mutant non-fluorescent GFP containing a total of ten copies of the myc epitope tag inserted into three distinct accessible positions [23]. The *cis*-regulatory sequences from *tef* were used to control expression. In addition, we generated lines with a *UASt-tef-myc* transgene for expression of a TEF fusion protein with ten tandemly repeated copies of the myc epitope tag at the C-terminus. To assess the functionality of these myc-tagged TEF versions, the transgenes were expressed in *tef* mutants (*tef*^z2-3455^/*tef*^z2-4169^), in case of *UASt-tef-myc* using the driver *bamP-GAL4-VP16*. The dodeca FISH assay indicated that regular chr3 segregation was largely or even completely restored by the expression of these transgenes (Fig 1F). Beyond the dodeca FISH assay, a second assay was used for confirmation of the functionality of TEF-sm_myc and TEF-myc. In this assay, we determined the DNA content of early spermatid nuclei microscopically [24]. Meiotic missegregation results in aneuploid spermatids and hence in increased variability of the DNA content in spermatid nuclei. In *tef* mutants, the increased variability of spermatid nuclear DNA content was detected as expected, and expression of TEF-sm_myc or TEF-myc reduced this variability to near control values (S1 Fig). These results demonstrate that TEF-sm_myc and TEF-myc are functional.

**Fig 2:**
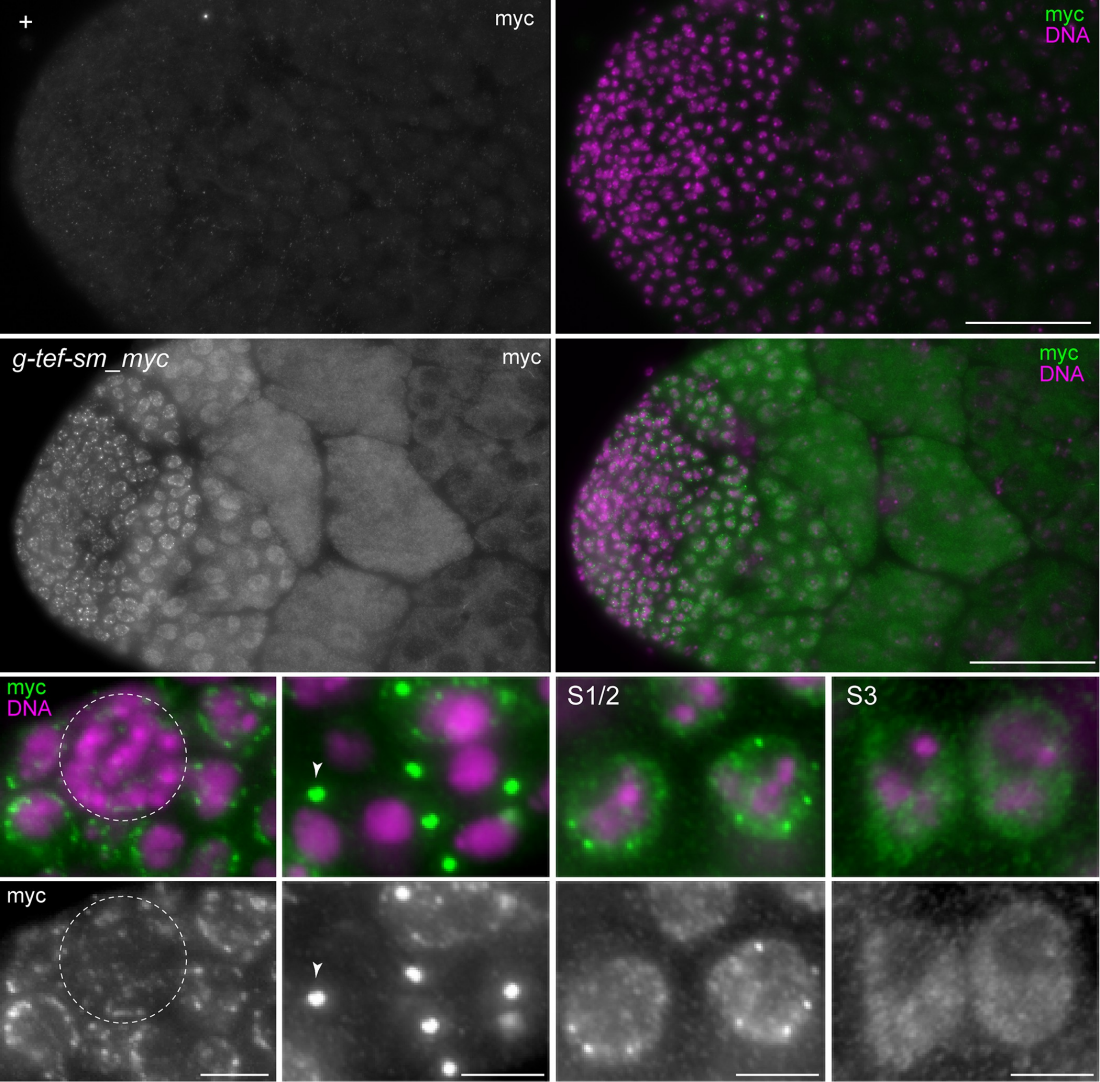
Expression and subcellular localization of TEF-sm_myc in testis. Flat preparations of testes isolated from either *w*^*1118*^ control (+; top row) or from *g-tef-sm_myc* II.1 males (*g-tef-sm_myc*; middle and bottom row) were immunolabeled with anti-myc and a DNA stain. Apical testis regions are shown (top and middle row), as well as high magnification views (bottom rows) with (from left to right) stem cells around the hub (dashed circle), spermatogonial cells during telophase with labeled spindle poles (arrowhead), early spermatocytes before chromosome territory formation (S1/2) and after completion of this process (S3). Scale bars = 50 μm (top and middle row) and 5 μm (bottom row).

To analyze expression pattern and subcellular localization of the myc-tagged TEF versions, we immunolabeled testis preparations from transgenic males with anti-myc. Both TEF-sm_myc and TEF-myc were readily detected (Figs 2 and S2B). In case of *g-tef-sm_myc* testes, indistinguishable results were obtained with two distinct transgene insertions (II.1 and III.1). The strongest anti-myc signals in these testes were observed in early S1/2 spermatocytes before chromosome territory formation within the cell nucleus (Fig 2). These strongest signals were granular at the nuclear periphery, where DNA staining was absent. In regions with DNA staining and in the nucleolus, anti-myc signals were low at most. In more advanced spermatocytes during the S3 stage, anti-myc signals were less granular and weaker, but still low or absent in the DNA staining regions and the nucleolus (Fig 2). In parallel with spermatocyte maturation, signal intensities decreased further. At the S5 stage, nuclear signals were no longer above background, although a weak diffuse signal persisted in the cytoplasm (Fig 2). TEF-sm_myc was also detected in cell types other than spermatocytes. It was clearly observed in germline stem cells (GSCs), during the gonial division cycles, and early but not late in the somatic cyst cell lineage (Fig 2). Germline cells during the pre-spermatocyte stages also displayed granular signals at the nuclear periphery during interphase (Fig 2). During meta-, ana- and telophase, spermatogonial cells had very strong signals on the spindle poles (Fig 2). TEF-sm_myc was low in hub cells, and apparently absent in epithelial and pigment cells of the testis sheet.

Based on transcript profiling data [25,26], maximal *tef* expression occurs in ovaries, while *tef* mRNA levels in testis and other tissues are far lower. Indeed, TEF-sm_myc was readily detectable in ovaries in the same pattern in two independent transgene insertion lines (S2A Fig). Signals in regions 1 and 2 of the germarium were low, but as in testis, centrosome staining in mitotically dividing cells was prominent (S2A Fig). In region 3 of the germarium, signals increased strongly in the cytoplasm of somatic follicle cells and to a lower degree in germline cells (S2A Fig). TEF-sm_myc remained readily detectable throughout oogenesis predominantly in the cytoplasm (S2A Fig). While these results reveal the presence of TEF protein in ovaries, the role of TEF in the female gonad remains unclear, as *tef* null mutant females do not appear to exhibit phenotypic abnormalities [8,9].

Compared to *g-tef-sm_myc*, testes of *bam>tef-myc* males displayed more transient anti-myc signals (S2B Fig). TEF-myc accumulation driven by *bamP-GAL4-VP16* started later, during the last spermatogonial division cycle. Anti-myc signals were considerably stronger in early spermatocytes (S1-S3) compared to those detected in *g-tef-sm_myc*. However, at later stages, anti-myc signals dropped precipitously also in *bam>tef-myc* testes. At the S5 stage and later, we were unable to detect TEF-myc above background. Transient anti-myc signals were also present in *bam>tef-myc* ovaries (S2C Fig). Subcellular localization of TEF-myc was similar to that of TEF-sm_myc, including the strong centrosomal signals during exit from M phase in both spermatogonial and oogonial cells (Figs 2 and S2).

**Fig 3:**
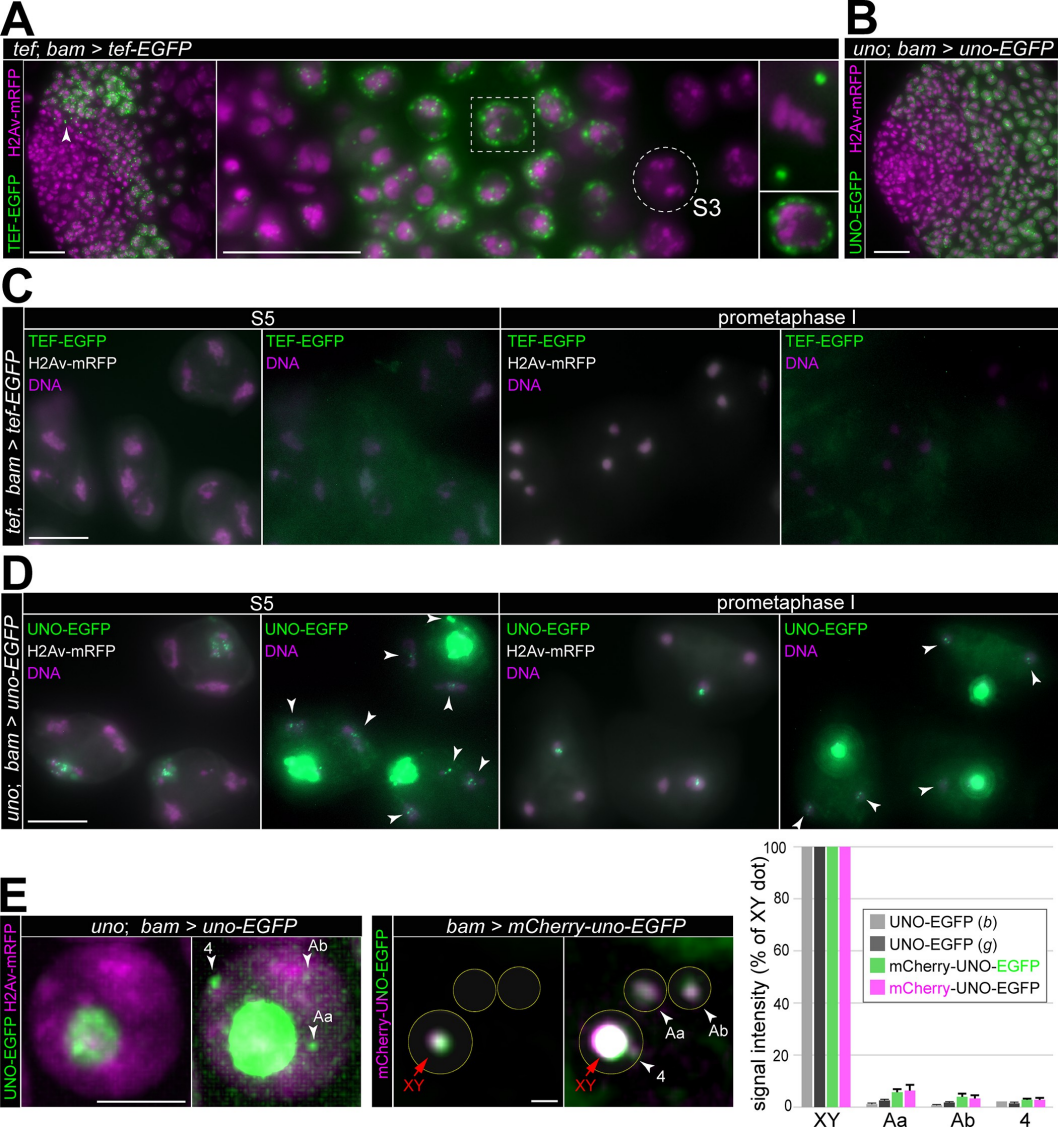
TEF-EGFP is not stably bound on autosomal bivalents until anaphase I in contrast to UNO-EGFP. (**A-D**) Analysis of genotypes, in which the expression of endogenous UNO and TEF was replaced by UNO-EGFP and TEF-EGFP, respectively, using *UASt* transgenes and *bamP-GAL4-VP16*. Apical testis regions (A,B) and late spermatocytes (C,D) are displayed. (**A**) TEF-EGFP is detectable only transiently in *tef* mutants with *bam*>*tef-EGFP*. During the last spermatogonial mitosis, TEF-EGFP is on spindle poles (arrowhead left panel, high magnification upper right panel) and in the following interphase granular around chromatin (equatorial section of nucleus boxed in middle panel displayed in lower right panel). At the S3 stage (circled in middle panel), TEF-EGFP is no longer detectable. (**B**) UNO-EGFP signals are maintained during spermatocyte maturation in *uno* mutants with *bam*>*uno-EGFP*. (**C**) TEF-EGFP signals are not detectable on bivalents in images acquired and displayed as those in (D). (**D**) UNO-EGFP signals on chrXY and autosomal bivalents. For a given stage, the same image is shown twice with distinct display settings, on the left without saturation of the UNO-EGFP signals on chrXY pairing region on the right with strong enhancement of the green channel to reveal the much weaker UNO-EGFP signals on autosomal bivalents (arrowheads). (**E**) Comparison of UNO amounts on chrXY and autosomal bivalents, respectively. Spermatocytes expressing His2Av-mRFP and UNO-EGFP in *uno* mutants (left) or mCherry-UNO-EGFP (right) were analyzed by time-lapse imaging. Representative still frames at S3 (left) and NEBD I (right) are shown twice, the second time with enhanced signal intensities to reveal the weak autosomal signals (arrowheads). The bar diagram displays relative UNO signal intensities at NEBD I with chrXY dot intensity set to 100%. The large autosomes with the stronger and weaker signals are designated as Aa and Ab, respectively. Mean signal intensities and standard deviation (s.d.) are displayed, n = 2 cells for *bam*>*uno-EGFP* in *uno* null mutants (UNO-EGFP (*b*)), 10 for *g-uno-EGFP* in *uno* null mutants (UNO-EGFP (*g*)), and 5 for *bam*>*mCherry-uno-EGFP* (mCherry-uno-EGFP, with color indicating green and red signals, respectively)). Scale bars = 20 μm (A,B), 10 μm (C,D) and 5 μm (E).

In part, expression pattern and localization of myc-tagged TEF versions expressed from *g-tef-sm_myc* and *bam>tef-myc* were unexpected. Despite several putative DNA-binding zinc fingers, the intracellular localization of these functional TEF versions was not obviously on chromatin. Most strikingly, they were not detected in late spermatocytes in contrast to the other AHC proteins (MNM, SNM and UNO), which remain detectable on bivalents until anaphase I [10,11]. Because the noticeable background levels that arose from anti-myc labeling may have concealed low levels of chromosomal TEF, we aimed for improved detection and therefore generated *UASt-tef-EGFP* lines. Analogous *UASt* transgenes for expression of MNM-EGFP, SNM-EGFP and UNO-EGFP with *bamP-GAL4-VP16* have previously permitted detection of these AHC proteins until anaphase I even on autosomal bivalents [11,24]. If TEF achieves physical linkage of autosomal homologs as an essential stoichiometric subunit in complexes with MNM, SNM and UNO, comparable autosomal signals are expected to be detectable in *bam>tef-EGFP* and *bam>uno-EGFP* testes, for example. Before comparing chromosomal EGFP signals in these genotypes, we confirmed that TEF-EGFP is also functional. Meiotic missegregation was largely prevented in *tef* mutants (*tef*^z2-3455^/*tef*^z2-4169^) by *bam>tef-EGFP* (S1 Fig). Microscopic analysis of this genotype revealed that the pattern of expression and the subcellular localization of TEF-EGFP was equivalent to that of TEF-myc in *bam>tef-myc* testes. The *bamP-GAL4-VP16* driven expression of TEF-EGFP started also in the last gonial division cycle with signals primarily detectable on the spindle poles during the final gonial mitosis (Fig 3A). In the following interphase, accumulation of TEF-EGFP occurred in fine granules at the periphery around the nuclear DNA staining (Fig 3A). However, already at the S3 stage, TEF-EGFP was no longer detectable (Fig 3A).

For comparison with UNO-EGFP, we repeated the analysis with testes from *uno* mutants with *bam>uno-EGFP* [11]. In this genotype, UNO-EGFP signals were most prominent in the nucleolus, remaining readily detectable throughout spermatocyte maturation from S1 until the latest S6 stage (Fig 3B and 3D), as reported [11]. Around the start of M I, the nucleolar UNO-EGFP foci were compacted into a single strong dot on the chrXY pairing region (Fig 3D), followed by disappearance during exit from M I [11]. In addition, considerably weaker, but often detectable UNO-EGFP dot signals were observed in autosomal chromosome territories and on autosomal bivalents at the start of M I (Fig 3D) [11]. In contrast, although TEF-EGFP signals in early spermatocytes were of comparable intensity to those resulting with UNO-EGFP (Fig 3A and 3B), they could not be detected in late spermatocytes of *bam>tef-EGFP* testes. With identical acquisition and display settings, neither the sex chromosome bivalent nor the autosomal bivalents had TEF-EGFP signals above background (Fig 3C).

The *tef* mutants with *bam>tef-EGFP* were also scrutinized by live imaging, as analogous analyses with *bam>uno-EGFP* in *uno* mutants had proven to be advantageous for distinguishing weak specific signals from random noise [11]. Specific signals but not noise maintain their spatial association with autosomes over time. However, also by time-lapse imaging, we were unable to detect autosomal TEF-EGFP in late spermatocytes and during M I even with the most strongly expressed *UASt-tef-EGFP* insertion.

We used *uno* mutants with *bam*>*uno-EGFP* and *His2Av-mRFP* for further characterization of the autosomal UNO-EGFP signals by live imaging. Autosomal UNO-EGFP signals started to become trackable in S3 spermatocytes (Fig 3E), once the relatively high uniform nuclear signals outside of the nucleolus had declined during S2. The number of UNO-EGFP signals per autosome could not be determined accurately because their weakness did not allow an unequivocal differentiation from background in all cells. Usually, we observed a few UNO-EGFP foci per autosome with those on chr4 most strongly focused (Fig 3E). The slow gradual chromosome condensation during the S6 stage was accompanied by focusing of the autosomal UNO-EGFP signals into one or two dots per bivalent. For a quantitative comparison of UNO-EGFP signal intensities on the chrXY bivalent with those on autosomal bivalents, we analyzed spermatocytes with these focused signals during NEBD I. Thereby, the amount of UNO on autosomal bivalents was found to be around 1–4% of that present on the chrXY pairing region (Fig 3E). Analogous quantification in spermatocytes expressing UNO-EGFP under control of the *uno cis*-regulatory region in an *uno* null mutant background gave comparable results (Fig 3E). Moreover, for reliable discrimination of weak autosomal signals from noise, we also included analyses with spermatocytes expressing a version of UNO with mCherry and EGFP fused at the N—and C-terminus, respectively (*bam*>*mCherry-uno-EGFP*) (Fig 3E and S2 Movie). While non-specific background signals in the green and red channels were not spatially correlated over time, the specific mCherry-UNO-EGFP signals were (S2 Movie). Quantification of the specific red and green signals was in excellent agreement and confirmed the results obtained in *uno* null mutants with either *bam*>*uno-EGFP* or *g-uno-EGFP* (Fig 3E).

In conclusion, while the UNO-EGFP dot signals on autosomal bivalents are considerably weaker than those on the sex chromosome bivalent, they can be detected above background at the onset of M I. In contrast, TEF-EGFP dot signals are not detectable on bivalents at this stage, even if the same *cis*-regulatory regions (*bam*>*UASt*) are used for expression.

### TEF recruits MNM onto polytene chromosomes of larval salivary glands

Although TEF does not associate stably with meiotic bivalents in amounts comparable to those of MNM, SNM and UNO, it might function in early spermatocytes during AHC establishment. It might contribute to the initial recruitment of the AHC proteins onto chromatin, as it includes three C2H2-type zinc fingers that are predicted to bind DNA. In contrast, MNM, SNM and UNO do not include known *bona fide* DNA-binding domains.

**Fig 4:**
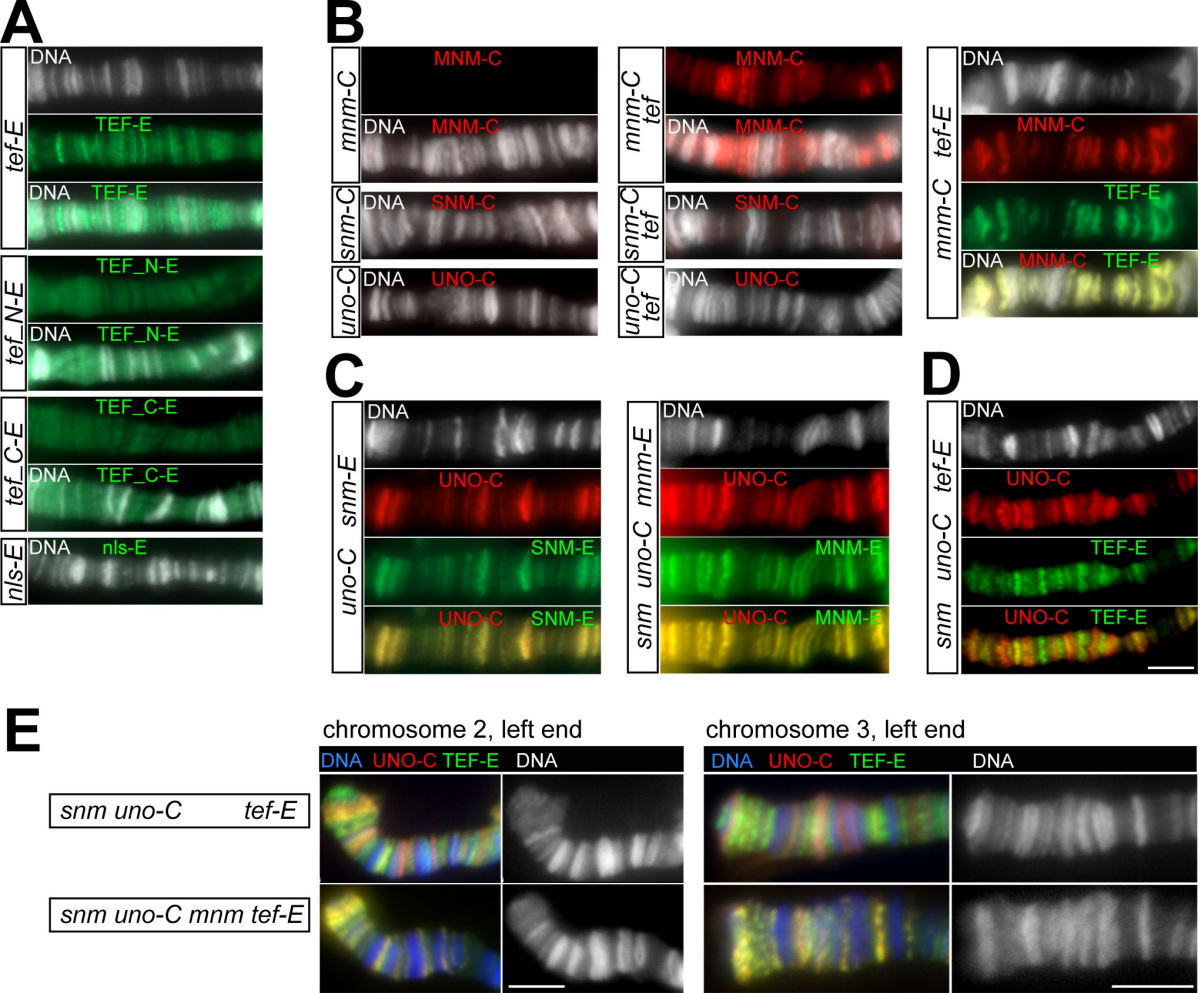
Binding of alternative homolog conjunction proteins to polytene chromosomes. (**A-E**) Squash preparations of larval salivary glands expressing various fluorescently tagged AHC proteins from *UASt* transgenes driven by *Sgs3-GAL4* for assessment of their binding to polytene chromosomes that were labeled with a DNA stain (DNA). (**A**) TEF binds efficiently to polytene chromosomes. While full-length TEF-EGFP (TEF-E) associates with specific sub-regions within interbands, the N-terminal (TEF_N-E) and C-terminal (TEF_C-E) halves of TEF, which include one and two zinc fingers, respectively, display only weak chromosome binding. No chromosome binding was observed for nls-EGFP (nls-E). (**B**) TEF recruits MNM to polytene chromosomes. MNM-mCherry (MNM-C), SNM-mCherry (SNM-C) and UNO-mCherry (UNO-C) do not associate with polytene chromosomes (left panel). However, after co-expression of TEF, MNM-C is recruited to polytene chromosomes, in contrast to SNM-C and UNO-C (middle panel). TEF-E and MNM-C are perfectly co-localized after co-expression (right panel). (**C**) UNO and SNM bind together to polytene chromosomes and can also recruit MNM. UNO-mCherry (UNO-C) and SNM-EGFP (SNM-E) are perfectly co-localized on polytene chromosomes after co-expression (left panel). MNM-EGFP (MNM-E) associates with polytene chromosomes when co-expressed with untagged SNM (SNM) and UNO-C, with a chromosomal localization that is identical to that of UNO-C (right panel). (**D**) TEF and SNM-UNO bind to distinct sub-regions on polytene chromosomes. TEF-E signals on polytene chromosomes are not co-localized with UNO-C signals after co-expression along with untagged SNM. (**E**) Co-localization of all four AHC proteins (SNM, UNO, MNM and TEF) on polytene chromosomes. UNO-C and TEF-E have a distinct banding pattern after co-expression with untagged SNM (as also shown in panel D) and an essentially identical banding pattern after co-expression with both untagged SNM and untagged MNM, indicating that MNM recruits, on the one hand, SNM-UNO-C to TEF-E binding sites and, on the other hand, TEF-E to SNM-UNO-C binding sites. Scale bars = 5 μm.

Therefore, we evaluated first whether TEF-EGFP can indeed bind to chromatin. For an evaluation of chromatin binding, we expressed *UASt-tef-EGFP* in salivary glands of larvae during the third instar wandering stage using *Sgs3-GAL4* [27]. TEF-EGFP was clearly detected in many interbands of the polytene chromosomes released from the nuclei in salivary gland squash preparations (Fig 4A). These chromosomal TEF-EGFP signals were not just present on the large autosomes (chr2 and chr3) but also on chrX, even though *tef* is not required for sex chromosome conjunction during male meiosis [8]. Control preparations from *Sgs3*>*nls-GFP* larvae did not display such chromosomal EGFP signals (Fig 4A). Absence of chromosomal nls-GFP signals was not a result of lower expression compared to TEF-EGFP. Quantification of nuclear EGFP signal intensities in whole-mount salivary gland preparations revealed comparable values in *Sgs3*>*nls-GFP* and *Sgs3*>*tef-EGFP* (S3 Fig). We conclude that TEF-EGFP can bind to chromatin in various distinct chromosomal regions.

TEF has multiple zinc fingers. One is N-terminal and the other two are C-terminal. To evaluate whether both the N- and the C-terminal zinc fingers might contribute to chromatin binding, we expressed *UASt* transgenes coding for either the N- or the C-terminal half of TEF fused to EGFP in larval salivary glands. Chromosomal EGFP signals in squash preparations of salivary glands from *Sgs3*>*tef-N-EGFP* and *Sgs3*>*tef-C-EGFP* were considerably weaker than those obtained with *Sgs3*>*tef-EGFP* (Fig 4A). Quantification of nuclear EGFP signals in whole-mount preparations indicated that the expression of the fragments TEF_N-EGFP and TEF_C-EGFP was higher than that of full-length TEF-EGFP (S3 Fig). Thus, we conclude that efficient binding of TEF to chromosomes appears to depend on a cooperation of its N- and C-terminal zinc fingers, which are in spatial proximity according to structure modeling by AlphaFold [28].

To address whether MNM, SNM and UNO also bind to chromatin like TEF, we made squash preparations with salivary glands isolated from larvae with *Sgs3-GAL4* and either *UASt-mnm-mCherry*, *UASt-snm-mCherry* or *UASt-uno-mCherry*. None of these three genotypes displayed substantial mCherry signals on polytene chromosomes (Fig 4B), indicating that MNM, SNM and UNO cannot bind directly to chromosomal DNA individually. However, if MNM-mCherry was co-expressed with untagged TEF (*Sgs3>tef*, *mnm-mCherry*), we readily observed chromosomal MNM-mCherry signals in many interbands (Fig 4B). Moreover, after co-expression of MNM-mCherry with tagged TEF-EGFP (*Sgs3>tef-EGFP*, *mnm-mCherry*), we observed a perfect co-localization of the chromosomal mCherry and EGFP signals (Fig 4B). We conclude that TEF can recruit MNM to chromatin. Consistent with an intimate interaction between TEF and MNM, we also observed mutual stabilization of TEF-EGFP and MNM-mCherry in salivary glands when co-expressed in salivary glands, according to quantifications of whole-mount preparations (S3 Fig).

While TEF recruited MNM-mCherry to polytene chromosomes, it did not recruit SNM-mCherry or UNO-mCherry (Fig 4B). Thus, we tested whether other combinations of AHC proteins without TEF might gain an ability to bind to polytene chromosomes. Interestingly, after co-expression of SNM-EGFP and UNO-mCherry we observed their perfect co-localization in many interbands of polytene chromosomes (Fig 4C). Comparable chromosome binding was not observed with the remaining pairwise combinations, MNM and UNO on the one hand, and MNM and SNM on the other hand, using the genotypes *Sgs3*>*mnm-EGFP uno-mCherry* and *Sgs3*>*mnm-EGFP snm* and *Sgs3*>*mnm snm-EGFP* for analysis. Our observations suggested that SNM and UNO form a complex (SU) and thereby gain the ability to bind to chromatin. Further support for SU complex formation was obtained from the quantification of signal intensities in whole-mount preparations (S3 Fig). SNM-E was observed to shift from the cytoplasm more into the nucleus upon co-expression with UNO-C (S3 Fig). Moreover, a considerable stabilization of UNO-mCherry was obtained after co-expression with SNM-EGFP (S3 Fig).

In spermatocytes, SNM and UNO are strictly co-localized with MNM, at least at the chrXY pairing region (Weber et al. 2020). Therefore, it appeared conceivable that not just TEF but also SU might be able to recruit MNM to chromatin. To address this possibility, we analyzed preparations made with *Sgs3*> *snm uno-mCherry mnm-EGFP* larvae. A precise co-localization of the red and green fluorescent chromosomal signals was observed (Fig 4C), confirming that SU recruits MNM to chromatin and arguing for the formation of complexes containing SNM, UNO and MNM (SUM).

To resolve whether TEF, on the one hand, and SU, on the other hand, bind to the same chromosomal sites on polytene chromosomes, we made preparations from *Sgs3*> *tef-EGFP snm uno-mCherry* larvae. The pattern of green fluorescent signals on the polytene chromosomes was not identical to that of the red fluorescent signals (Fig 4D and 4E), indicating that TEF and SU have distinct chromatin binding specificities. However, the differences between the patterns of green and red fluorescent chromosomal signals were largely eliminated if MNM was also expressed along with TEF, SNM and UNO, as in preparations with *Sgs3*>*snm uno-mCherry mnm tef-EGFP* larvae (Fig 4E). In these preparations, all the chromosomal locations with fluorescent signals that were almost exclusively red or green after co-expression of TEF-EGFP, SNM, and UNO-mCherry, displayed fluorescent signals also after co-expression of MNM with these proteins, but their color was changed towards yellow (Fig 4E). These findings are readily explained if MNM, after recruitment to chromatin by SU, can recruit TEF, and if MNM, after recruitment to chromatin by TEF, can recruit SU. Thus, we suggest that the AHC proteins SNM, UNO, MNM and TEF have the ability to form chromosomal complexes, which contain all four proteins (SUMT).

Beyond interactions with AHC proteins and chromosomes, ectopic expression in salivary glands exposed another trait in case of MNM. MNM’s subcellular localization was found to depend on the fluorescent tag and the expression level. *Sgs3-GAL4* drives strong expression in basal cells, which are in the distal part of the salivary gland, and weak expression in transition cells, which are proximal to the duct, and expression in both cell types increases during the third larval instar [27]. Strong expression of MNM-EGFP in basal cells of late larvae was accompanied by the formation of intranuclear droplets (S3 Fig), perhaps reflecting liquid-liquid phase separation [29]. In contrast, with MNM-mCherry, droplets were formed to a far lower extent (S3 Fig). However, after co-expression of MNM-mCherry with TEF-EGFP, the two strictly co-localized proteins shifted with increasing expression from a localization in bands associated with polytene chromosomes towards droplets in between chromosomes (S3 Fig). When TEF-EGFP was expressed alone, we never observed droplet formation (S3 Fig). These observations suggested that MNM might have a tendency to undergo phase separation that is enhanced by EGFP but not mCherry, because the former but not the latter forms weak homodimers. Accordingly, TEF-EGFP might contribute to phase separation of MNM-mCherry because its tight binding to MNM-mCherry brings in a weakly dimerizing EGFP moiety.

### TEF binds to MNM and indirectly to SNM and UNO

**Fig 5:**
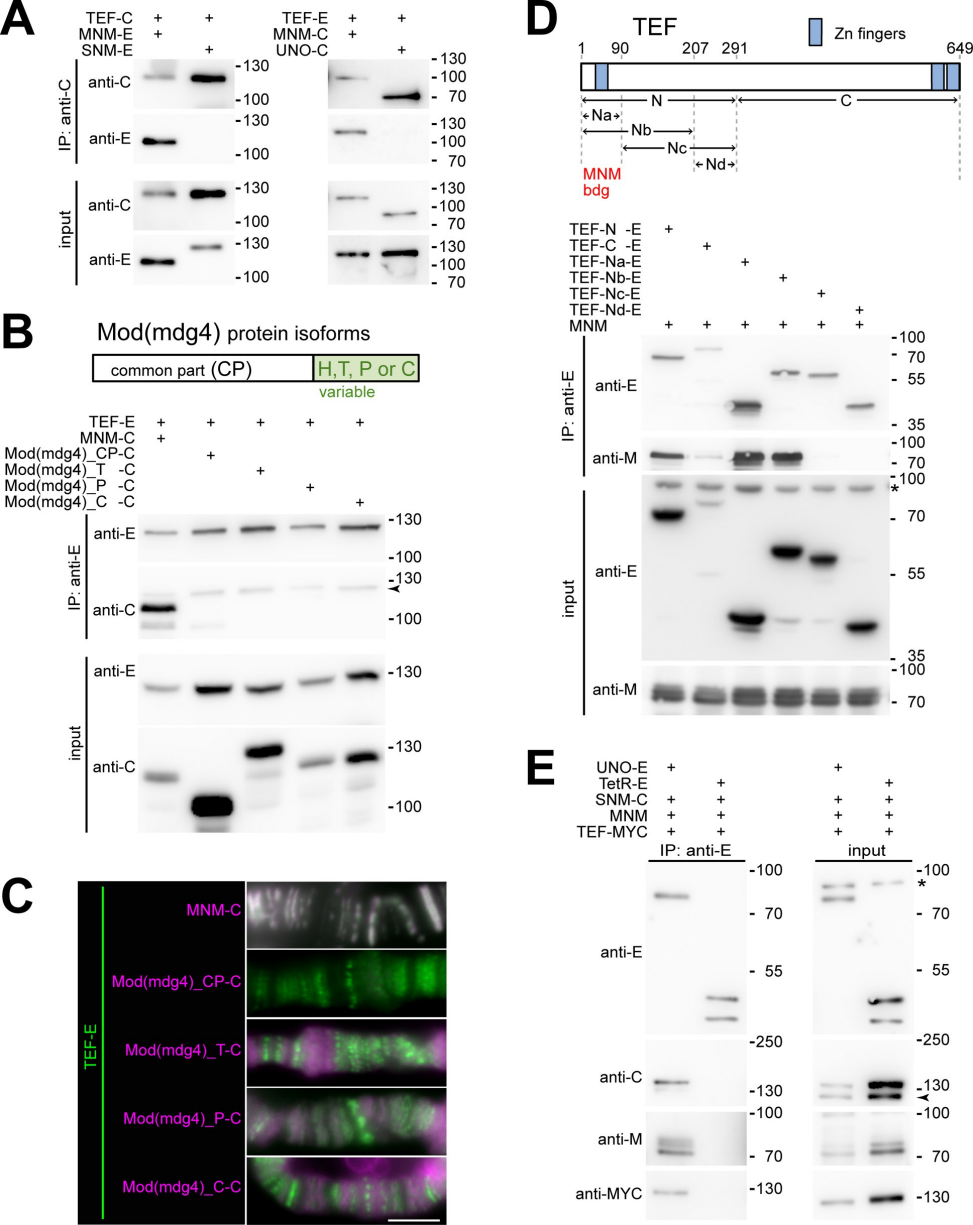
TEF protein interactions with AHC proteins revealed by co-immunoprecipitation experiments. (**A,B,D,E**) S2R+ cells were transfected for transient co-expression of AHC proteins, Mod(mdg4) protein isoforms and nls-tetracycline-repressor (TetR) for control as indicated. The expressed proteins were tagged with either EGFP (-E), mCherry (-C), myc epitope (-MYC) or untagged. After extract preparation, antibodies against EGFP (anti-E) or mCherry (anti-C) were used for immunoprecipitation. Input extracts and immunoprecipitated proteins were analyzed by immunoblotting with anti-E, anti-C, anti-MYC and anti-M, an antibody against the region present in all Mod(mdg4) protein isoforms including MNM. A non-specific band is marked (*), as well as bands (arrowheads), which re-appeared when re-probing with another antibody. (**A**) TEF-C co-precipitates MNM-E but not SNM-E, and TEF-E is co-precipitated with MNM-C but not with UNO-C. (**B**) TEF-E co-precipitates MNM-C but not the other Mod(mdg4) isoforms CP, T, P and C. As indicated (top), all Mod(mdg4) isoforms share an N-terminal common part (CP) that is followed by distinct isoform-specific C-terminal regions. (**C**) On polytene chromosomes from larval salivary glands, TEF-EGFP (TEF-E) is perfectly co-localized with MNM-mCherry (MNM-C) but not with the other Mod(mdg4) isoforms CP, T, P and C fused to mCherry (-C). The perfect overlap of the green TEF-EGFP and magenta MNM-mCherry signals results in shades of grey in the top rgb image. Scale bar = 5 μm. (**D**) The N-terminal region of TEF mediates MNM binding. MNM was co-expressed with TEF-E or with the EGFP tagged TEF fragments schematically shown on top. The Na fragment is sufficient for co-precipitation of MNM. (**E**) All four AHC proteins (SNM, UNO, MNM and TEF; abbreviated SUMT) were detected after co-expression in UNO-E immunoprecipitates. SUMT complex formation reflects specific associations as revealed by a control experiment, in which UNO-E was replaced with TetR-E.

To confirm the interactions between TEF and the other AHC proteins indicated by our microscopic analyses with larval salivary glands, we performed co-immunoprecipitation (co-IP) experiments. After transient co-expression of TEF-mCherry with MNM-EGFP in S2R+ cells and immunoprecipitation of TEF-mCherry, co-precipitation of MNM-EGFP was readily detected (Fig 5A). Efficient co-precipitation of TEF and MNM was also observed with swapped tags and immunoprecipitation of MNM instead of TEF (Fig 5A). In contrast, in parallel experiments, we were unable to detect co-precipitation of TEF with SNM or UNO (Fig 5A). These results fully agree with the observations in salivary glands, where TEF recruited MNM to polytene chromosome bands but not SNM or UNO.

MNM is encoded by the complex locus *mod(mdg4)*, which expresses more than 30 protein isoforms [10]. These isoforms, including MNM, share an N-terminal common part (CP) followed by an isoform-specific unique C-terminal region, encoded by differentially spliced exons [10,12,13]. To address whether TEF binds exclusively to MNM or also to other Mod(mdg4) isoforms, we performed additional co-IP experiments. For these, we generated plasmids for expression of the Mod(mdg4) isoforms T, P and C (Fig 5B). The T isoform (also designated as 67.2) represents the most extensively characterized Mod(mdg4) product. It is known to contribute to the function of the gypsy insulator along with Su(Hw) and CP190 [30,31]. The C and P isoforms are likely expressed in the testis [26]. Moreover, we also made a construct for expression of the shared N-terminal common part (CP) of the Mod(mdg4) proteins (without any of the isoform-specific C-terminal extension). Our co-IP experiments demonstrated that TEF-EGFP binds exclusively to MNM-mCherry. The other mCherry fusions (CP, T, P and C) were not co-precipitated by TEF-EGFP (Fig 5B). These findings were corroborated by microscopic analyses after co-expression of TEF-EGFP and these Mod(mdg4) in salivary glands. In contrast to the perfect co-localization of TEF-EGFP and MNM-mCherry on polytene chromosomes (Figs 4B and 5B), TEF-EGFP did not co-localize with Mod(mdg4)_CP, _T, _P and _C fused to mCherry (Fig 5C). While Mod(mdg4)_CP-mCherry was clearly expressed in salivary glands, as evident from whole-mount preparations (S4 Fig), squash preparations indicated that its binding to polytene chromosomes was marginal (Fig 5C). The mCherry fusions of the Mod(mdg4) isoforms T, P and C did bind to polytene chromosomes, as demonstrated previously by immunostaining in case of the T and C isoforms [13]. However, the pattern of polytene chromosome association of these isoforms was evidently distinct from that of TEF-EGFP (Fig 5C).

To identify the region of TEF that binds to MNM, we performed co-IP experiments after co-expression of MNM-mCherry with TEF fragments fused to EGFP. The results of these co-IP experiments revealed that the N-terminal 90 amino acids of TEF are sufficient to mediate binding to MNM (Fig 5D). Additional co-IP experiments indicated that TEF has the ability to dimerize or oligomerize (S5 Fig).

In salivary glands, MNM induced a co-localization of TEF-EGFP with SNM-UNO-mCherry, arguing for the formation of complexes containing all four AHC proteins (SUMT). For further confirmation that SUMT complexes can be formed, we performed a co-IP experiment after transient expression of all four AHC proteins (SNM-mCherry, UNO-EGFP, MNM and TEF-myc). Indeed, UNO-EGFP co-precipitated the three other AHC proteins readily (Fig 5E). In contrast, in a control experiment, in which an EGFP fusion of the tetracycline repressor was precipitated instead of UNO-EGFP, we did not detect co-precipitation of the other AHC proteins (Fig 5E). In conclusion, our results demonstrate that SUMT complexes can be formed.

### Overexpression of SUM or SUMT in spermatocytes inhibits chromosome territory formation and normal meiotic chromosome segregation

TEF was found to be sufficient for recruitment of SUM (SNM, UNO and MNM) to chromosomes in salivary glands (Fig 4E). Hence, the recruitment of SUM to autosomal bivalents in spermatocytes might also be promoted by TEF. Moreover, the transient presence of endogenous TEF in early spermatocytes, as indicated by *g-tef-sm_myc* (Fig 2), might limit SUM recruitment and prevent excess loading. Accordingly, TEF overexpression in spermatocytes is predicted to increase the amount of SUM on autosomal bivalents. To evaluate this possibility, we analyzed the effect of TEF overexpression (*bam*>*tef*) on the intensity of autosomal UNO-EGFP dots in spermatocytes expressing this fluorescently tagged AHC protein under control of the *uno cis*-regulatory sequences [11]. The intensity of autosomal UNO-EGFP dots at the S6 stage was not evidently increased after TEF overexpression, but due to the difficulties of quantifying these weak autosomal signals precisely, a minor effect could not be ruled out.

**Fig 6:**
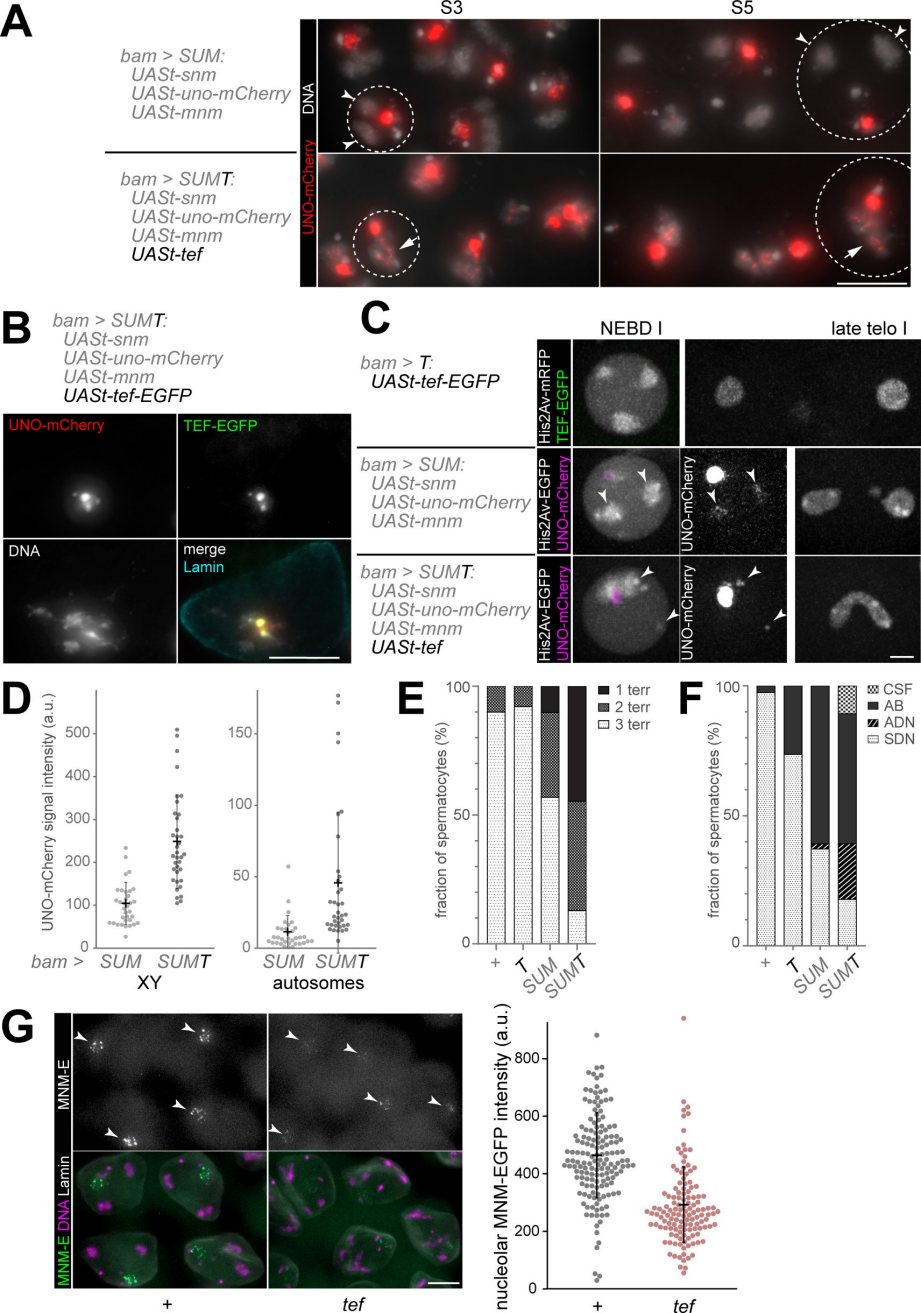
Co-overexpression of SNM, UNO and MNM has a more pronounced effect on spermatocytes when TEF is overexpressed as well. (**A-F**) Overexpression of AHC proteins in spermatocytes. *bamP-GAL4-VP16* was used for expression of the indicated *UASt* transgenes. (**A**) Spermatocytes expressing SUM (top) or SUMT (bottom) including UNO-mCherry from testis squash preparations. Some of the nuclei are indicated (dashed circle). After SUM expression, the territories of the sex chromosomes and of the large autosomes (arrowheads) are separated apart. In contrast, a single large territory with sex chromosomes and autosomes (arrows) is present after SUMT expression. (**B**) Spermatocyte expressing SUMT during the S5 stage with TEF-EGFP partially co-localized with UNO-mCherry. In addition to DNA staining, the squash preparation was also labeled with anti-Lamin. (**C-F**) Analyses after time-lapse imaging. (**C**) Still frames from spermatocytes expressing TEF-EGFP (top), SUM (middle) or SUMT (bottom) at the onset of NEBD I and 75 minutes later early interkinesis. For SUM and SUMT, the images at NEBD I onset are displayed twice, on the left with nucleolar UNO-mCherry shown without signal saturation and on the right enhanced UNO-mCherry signals to reveal the weak autosomal signals (arrowheads). (**D**) Chromosome-associated UNO-mCherry signals within the nucleolus (XY) and outside the nucleolus (autosomes) were quantified at NEBD I in spermatocytes expressing SUM or SUMT. n = 34 (SUM) and 36 (SUMT) spermatocytes derived from multiple cysts (5 for SUM and 6 for SUMT). Mean +/- s.d. is indicated as well. (**E**) The number of major chromosome territories was scored around NEBD I. Number of analyzed spermatocytes = 40 (+), 89 (T), 79 (SUM) and 87 (SUMT). The spermatocytes were derived from multiple cysts, i.e., from 5 (+), 12 (T), 10 (SUM) and 13 (SUMT). (**F**) The success of chromosome segregation during exit from M I was scored. Spermatocytes generating apparently normal symmetric daughter nuclei (SDN), clearly abnormal asymmetric daughter nuclei of unequal size (ADN), anaphase bridges (AB), or displaying a complete chromosome segregation failure (CSF) were counted. Number of analyzed spermatocytes = 39 (+), 19 (T), 51 (SUM) and 56 (SUMT). The spermatocytes were derived from multiple cysts: 4 (+), 4 (T), 6 (SUM) and 7 (SUMT). (**G**) *tef* mutant spermatocytes have reduced amounts of nucleolar MNM. EGFP signal intensity in nucleoli (arrowheads) was quantified microscopically in spermatocytes expressing *hs-mnm-EGFP* in either a *tef* ^+^ (+) or a *tef* mutant (*tef*) background. Basal *hs-mnm-EGFP* expression was analyzed with testis squash preparations after immunolabeling with anti-Lamin and a DNA stain. Nucleolar MNM-EGFP intensities (a.u.) in S5/6 spermatocytes are indicated by dots. Mean and s.d. are also displayed. n = 154 (+) and 131 (*tef*). Scale bar = 10 μm (A,B,G) and 4 μm (C).

TEF overexpression might not have increased UNO-EGFP amounts on autosomal bivalents because the endogenous expression of one or several of the SUM proteins might not be high enough to permit excess loading. To address this possibility, we analyzed the effects of spermatocyte-specific overexpression of SUM and SUMT, respectively. Overexpression was driven by *bamP-GAL4-VP16* in combination with *UASt* transgenes coding for untagged AHC proteins, except for UNO, which had a C-terminal mCherry extension. In *bam*>SUM spermatocytes, UNO-mCherry signals in the nucleolus were very strong, but the large autosomal territories did not appear to display evidently increased UNO-mCherry signals (Fig 6A, top). In *bam*>SUMT spermatocytes, UNO-mCherry signals were not only very high in the nucleolus, but additional foci were scattered on chromatin surrounding the nucleolus (Fig 6A, bottom), most likely primarily on autosomal chromatin. As chromosome territory formation was severely inhibited in *bam*>SUMT spermatocytes (Fig 6A, bottom), an unequivocal assignment of all the finer dots to autosomal bivalents was impossible.

If TEF acted merely by directing SUM to autosomes, the apparent increase in autosomal UNO-mCherry in *bam*>SUMT spermatocytes should have occurred at the expense of nucleolar UNO-mCherry. However, compared to *bam*>SUM, *bam*>SUMT spermatocytes appeared to have increased rather than decreased nucleolar UNO-mCherry signals (Fig 6A). For a quantitative corroboration of the observations made with the testis squash preparations, we performed time-lapse imaging and analyzed UNO-mCherry signals at the onset of NEBD I, a precisely defined developmental stage (Fig 6C and 6D). Quantification of UNO-mCherry signal intensities confirmed that both the non-nucleolar, signals and the nucleolar signals were significantly increased in *bam*>SUMT compared to *bam*>SUM (4.3 and 2.4 fold, respectively) (Fig 6D). These results argue against the notion that TEF functions exclusively by re-directing SUM complexes away from the sex chromosome bivalents onto autosomal bivalents. Instead, the TEF overexpression in *bam*>SUMT spermatocytes augmented the levels of UNO-mCherry (and most likely also of SNM and MNM) on all bivalents at the start of M I. Yet, TEF appeared to impose some bias in chromosomal SUM recruitment toward autosomes, as indicated by the quantitative comparison of UNO-mCherry signal intensities in *bam*>SUM and *bam*>SUMT spermatocytes. Compared to the former genotype, the latter was characterized by an increase in UNO-mCherry signal intensity that was 1.8-fold greater on autosomal bivalents than in the nucleolus (Fig 6D).

Beyond diverting some UNO-mCherry away from sex chromosomes onto autosomal chromosomes, TEF resulted primarily in a strong increase in the total amounts of chromosomal UNO-mCherry in our SUMT overexpression experiments. Thus, TEF appears to stabilize the SUM proteins in spermatocytes, as also observed in salivary glands. Conversely, loss of TEF might result in lower SUM protein levels in spermatocytes. To evaluate this possibility, we compared the level of nucleolar MNM-EGFP in spermatocytes with and without *tef* function. The signals resulting from basal expression of *hs-mnm-EGFP*, a transgene controlled by *hsp70 cis*-regulatory sequences [10], were analyzed in late spermatocytes (around the S5 to S6 transition). Nucleolar MNM-EGFP was found to be around 40% lower in *tef* null mutants compared to controls (Fig 6G). This finding is again at odds with the hypothesis that TEF functions exclusively to redirect SUM away from sex chromosomes onto autosomes because nucleolar MNM-EGFP is predicted to be increased in *tef* mutants according to this hypothesis and not decreased as observed. However, the result supports the notion that TEF stabilizes the SUM proteins. Vice versa, we also found that SUM proteins had a TEF stabilizing effect. While TEF-EGFP was no longer detected in late S5 spermatocytes in *bam*>TEF-EGFP spermatocytes (Fig 3C), it could be clearly detected at this stage in *bam*>SUMT spermatocytes expressing TEF-EGFP in addition to UNO-mCherry, which were partially co-localized in some of the chromosomal dots (Fig 6B), consistent with SUMT complex formation. These TEF-EGFP signals in late spermatocytes were far weaker than those in early spermatocytes, indicating a limited stabilization of TEF-EGFP by SUM co-expression.

Time-lapse imaging of *bam*>SUM and *bam*>SUMT spermatocytes also confirmed the inhibitory effect of AHC protein overexpression on chromosome territory formation previously suggested by the squash preparations (Fig 6A). For quantification, we analyzed spermatocytes around NEBD I (Fig 6C and 6E). Three major chromosome territories are usually detected in normal spermatocytes at this stage [7]. After time-lapse imaging and application of stringent criteria, 90% of the control spermatocytes displayed three completely separated major territories and the remainder two (Fig 6E). Similar numbers were obtained for *bam*>*tef-EGFP* spermatocytes (Fig 6E and S3 Movie). However, in *bam*>SUM spermatocytes, the number of cells with three completely separated territories was reduced (Fig 6E and S4 Movie). Beyond cells with only two major territories, we also detected some with only one territory (Fig 6E). Finally, in *bam*>SUMT spermatocytes, the reduction in territory numbers was even more severe (Fig 6E and S5 Movie). We conclude that chromosome territory formation in spermatocytes is inhibited by SUM and more dramatically by SUMT overexpression.

Failure of normal chromosome territory formation before M I was shown to cause chromosome missegregation during M I [7]. The territory formation failure in *bam*>SUM and *bam*>SUMT spermatocytes was also accompanied by segregation defects (Fig 6C and 6F; and S4 and S5 Movies). However, compromised chromosome segregation during M I was detected even more frequently than territory formation failure. A substantial number of *bam*>SUM spermatocytes with normal territory organization at the onset of M I displayed chromosome bridges during anaphase I, presumably reflecting an inability to eliminate excess conjunction between homologs with sufficient efficiency. Similarly, a modest increase in the frequency of transient anaphase bridges was also detected in *bam*>*tef-EGFP* spermatocytes (Fig 6F), in which territory formation was not affected.

In conclusion, increased levels of SUM compromised normal chromosome territory formation and regular chromosome segregation during M I, most likely by inducing excess conjunction between homologs and ectopic conjunction between non-homologous chromosomes. Moreover, the presence of increased levels of TEF promoted a further increase in chromosomal SUM levels and a stronger failure of territory formation and chromosome segregation during M I.

### Overexpression of SUM and SUMT during mitotic cell proliferation results in ectopic chromosome conjunction and anaphase defects

While transcripts of *uno*, *mnm* and *tef* can be detected in various cell types other than spermatocytes, *snm* mRNA appears to be present exclusively in testes. Thus, in wild type, the full complement of all known AHC proteins is present likely only in spermatocytes. Forcing cell types other than spermatocytes to express all the known AHC proteins appeared of interest regarding the potential existence of additional, presently unknown spermatocyte-specific factors essential for AHC. If no additional spermatocyte-specific factors beyond those already known were required for AHC, ectopic SUMT expression might induce AHC during mitotic division. In *D*. *melanogaster*, pairing of homologs is known to occur efficiently in essentially all somatic cell types during interphase but homolog pairing is disrupted again at the start of somatic mitoses [32,33]. However, if SUMT expression was sufficient to cause AHC in somatic cells, homolog pairing might remain stable during entry into mitosis and thereby compromise chromosome bi-orientation. Consequential chromosome missegregation would result in aneuploidy and hence in non-viable daughter cells frequently. Accordingly, if SUMT expression causes AHC but not other adverse effects, it is predicted to be toxic exclusively in mitotically proliferating cells, while it should not affect terminally differentiated post-mitotic cells. Similarly, as TEF is not required for sex chromosome conjunction in spermatocytes, ectopic SUM might be sufficient to induce conjunction and consequential missegregation of sex chromosomes during mitotic divisions of male somatic cells.

**Fig 7:**
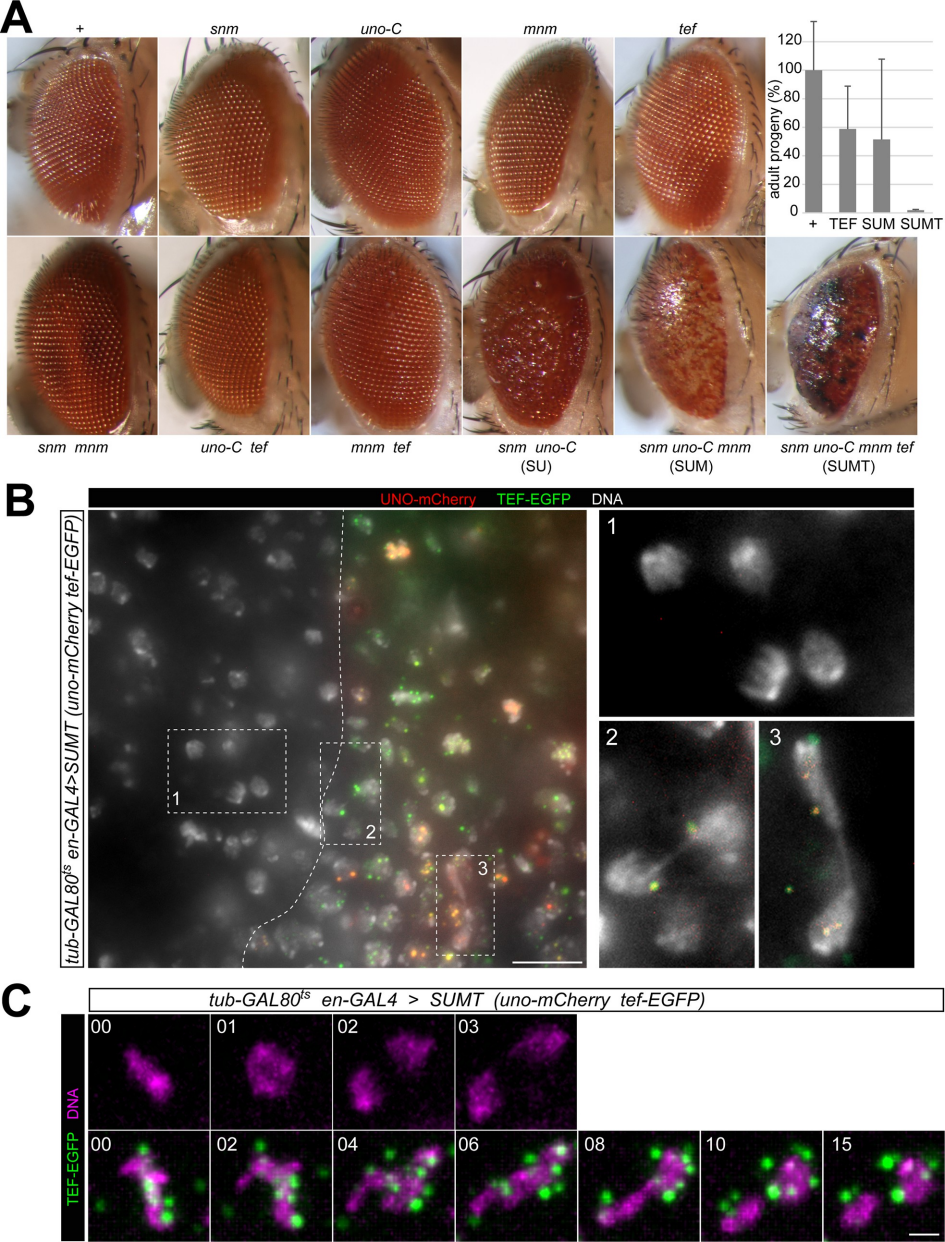
Ectopic expression of SUMT in somatic cells results in chromosome conjunction during mitosis. (**A**) Toxicity of ectopic AHC protein expression during eye development. Eyes of adult progeny with *GMR-GAL4* alone (+) or in combination with *UASt* transgenes coding for the indicated AHC proteins are displayed. Bars represent mean number of adult progeny with the indicated genotype that was obtained. Progeny number obtained in control crosses (+) was set to 100%. n = 5 crosses, whiskers indicate s.d. (**B**) Ectopic SUMT expression in wing imaginal discs. Untagged SNM and MNM were co-expressed with UNO-mCherry and TEF-EGFP in the posterior compartment during 16 hours of incubation at 29°C before fixation and DNA staining. A region from the wing pouch is shown with the compartment boundary (dashed line) separating non-expressing control cells in the anterior compartment (left) from SUMT-expressing cells in the posterior compartment (right). While normal anaphase and telophase figures are present in the anterior compartment (box 1), those in the posterior compartment are abnormal with chromosome bridges (boxes 2 and 3). (**C**) SUMT was expressed in imaginal wing discs as described above (B). Dissected wing imaginal discs were stained with a live DNA stain and analyzed by time-lapse imaging at 1 min intervals. Representative cells from the anterior control compartment (top) and from the posterior SUMT-expressing compartment (bottom) are shown during exit from mitosis. Time (min) with t = 0 representing the last metaphase frame is indicated. Note that UNO-mCherry signals are occluded by the intense red fluorescent DNA stain. Scale bars = 10 μm (B) and 2 μm (C).

To assess the toxicity of forced SUM and SUMT expression in proliferating and non-proliferating cell types, we used various GAL4 drivers (*ey-*, *sev-*, *GMR-*, *Act5C-*, *alphaTub84B-* and *en-GAL4*). Ectopic SUMT expression was found to be severely toxic. It caused substantial or complete developmental lethality depending on the driver (S1 Table). However, SUMT toxicity did not appear to be restricted to proliferating cells, as suggested by the results obtained with *GMR-GAL4*, which drives expression primarily in post-mitotic cells posterior of the morphogenetic furrow in eye imaginal discs during larval development and in the adult eye. SUMT expression driven by *GMR-GAL4* caused almost complete developmental lethality, and the few adult escapers had severely abnormal eyes (Fig 7A). In proliferating cells, ectopic SUMT expression appeared to be toxic as well, as indicated by the findings obtained with *ey-GAL4*, a driver primarily active in proliferating eye imaginal disc cells. With *ey-GAL4*, SUMT resulted in 83% lethality (S1 Table). Similarly, SUMT expression with the drivers *Act5C*-, *alphaTub84B*- and *en-GAL4* that are active in many cells already during early embryogenesis resulted in complete lethality (S1 Table).

Beyond ectopic SUMT expression, that of SUM and SU was also associated with toxicity, although to a lower degree (S1 Table). With GMR-GAL4, for example, SUMT reduced the number of adult progeny to 2% of control values, while the reduction obtained with SUM or TEF was only up to 49% and 41% of control values and hence substantially more moderate (Fig 7A). The adverse effects of ectopic SUM expression were observed to a comparable extent in males and females. Adult escapes displayed severe eye abnormalities (Fig 7A). Ectopic expression of AHC proteins individually did not have obvious adverse effects, except for TEF, which caused some developmental lethality (Fig 7A and S1 Table). Similarly, the pairs SNM-MNM, MNM-TEF and TEF-UNO-mCherry did not cause abnormal eye phenotypes after ectopic expression with *GMR-*, *ey*- and *sev*- *GAL4* (Fig 7A and S1 Table).

In conclusion, the toxic effects of AHC proteins were maximal when all were co-expressed together (SUMT). The combinations SU and SUM, which bind to chromatin according to the analyses with salivary glands, had milder effects. TEF individually, which also binds to chromatin, had even milder effects, also in combination with MNM. In contrast, other AHC proteins individually and in combinations (SNM, UNO-mCherry, MNM, and the pairs SM and UT), which did not display chromosome binding in larval salivary glands, did not have adverse effects.

Even though toxic effects of ectopic SUM and SUMT expression might arise also in non-proliferating cells, it remained a possibility that ectopic chromosome conjunction and consequential mitotic defects might contribute to their toxicity in proliferating cells. While the corresponding mitotic defects might appear already during the first mitosis after the onset of ectopic expression, toxicity in non-proliferating cells did not develop rapidly according to our observations in larval salivary glands. To characterize effects on mitosis early after the onset of ectopic SUM or SUMT expression, we started first with analyses in early embryos. In embryogenesis, paternal *UASt* transgenes that are crossed into oocytes containing maternally contributed GAL4-VP16 start to be expressed during the major wave of genome activation accompanying cellularization, i.e., during interphase of the embryonic division cycle 14. Therefore, we analyzed embryos microscopically after fixation during the stages of interphase 14 (I14) and the following mitotic division (M14). SUM or SUMT were expressed from *UASt* transgenes coding for untagged AHC proteins except for UNO, which had a C-terminal mCherry extension. The mCherry signals were detectable already during I14 (S6A Fig). In case of SUM expression, signals were restricted to the nucleolus. During M14, they were low and diffuse throughout the cells. In the following I15, signals were first present throughout the nucleus (S6A Fig), concentrating later again in the nucleolus. After SUMT expression, UNO-mCherry signals were also nucleolar during I14 (S6A Fig). However, during M14, signals displayed a localization that was clearly distinct from those in SUM embryos. In SUMT embryos, many bright dots were displayed during M14 instead of the weak diffuse signals observed in SUM embryos. While some of the mitotic UNO-mCherry dots were on chromosomes in SUMT, others did not appear to be chromosome-associated. After dot disappearance during exit from M14, re-accumulation of UNO-mCherry signals during I15 occurred in SUMT as described above for SUM. Abnormal mitotic figures during M14 were not or very rarely detected in SUM and SUMT embryos.

Our comparison of SUM and SUMT embryos suggested that TEF might trigger formation of AHC dots that are in part chromosome-associated but only during mitosis. For further evaluation of this possibility, we performed analyses with embryos expressing SUMT with distinct fluorescent tags on both TEF (TEF-EGFP) and UNO (UNO-mCherry) (S6B Fig). For comparison, we also analyzed embryos expressing only TEF-EGFP. In these latter embryos, green signals were detected in intranuclear dots during I14. During M14, the dots were in part chromosome associated (S6A Fig). Moreover, during M14, TEF-EGFP was also enriched on centrosomes, most strongly during exit from mitosis (S6A Fig). In case of SUMT embryos expressing TEF-EGFP and UNO-mCherry, the green and red signals were not co-localized during I14 (S6B Fig). UNO-mCherry was in the nucleolus and TEF-EGFP in fine nuclear dots (S6B Fig). Interestingly, however, during M14, the two tagged AHC proteins were strictly co-localized in dots that were more prominent than the TEF-EGFP dots during I14 (S6B Fig). Overall, our microscopic analysis with embryos suggested that the formation of SUMT complexes is regulated in a cell cycle-dependent manner, with M phase stimulating SUMT complex formation. For causing ectopic chromosome conjunction, these complexes presumably arise too late, i.e., after chromosome condensation at the onset of mitosis has disrupted somatic homolog pairing and the non-homologous associations in the chromocenter.

Ectopic SUM and SUMT expression might have more severe effects, if the time available for AHC protein accumulation is longer than during embryonic cell cycle 14, which lasts only for 1–2 hours. For analyses during longer cell cycles, we performed experiments with wing imaginal discs at a stage when progression through a cell division cycle takes about 16 hours on average [34]. To regulate SUMT expression, we used *en-GAL4* in combination with *tub-GAL80*^*ts*^. During initial development, a permissive low temperature (18°C) was applied, allowing GAL80^ts^ to block SUMT expression. Eventually, wing imaginal discs were dissected from third instar wandering stage larvae that had been exposed to 29°C during the 16 hours preceding fixation. This temperature results in induction of SUMT expression specifically within the posterior compartment. Among the ectopically expressed SUMT proteins, UNO and TEF were tagged with mCherry and EGFP, respectively. In fixed preparations (Fig 7B), we detected a distinct localization of UNO-mCherry and TEF-EGFP in some interphase cells, in nucleolus and intranuclear dots, respectively, as observed before in embryos. However, other cells displayed an obvious co-localization in intranuclear dots already during interphase. Moreover, mitotic figures were abnormal and revealed extensive chromosome lagging, indicating that SUMT expression compromises anaphase (Fig 7B). These mitotic abnormalities were detected exclusively in the posterior compartment. Within the wing pouch region, there were between 1–3 abnormal late mitotic figures in the posterior compartment (n = 10 imaginal discs). In contrast, in the anterior compartment, which did not express SUMT and thus served as internal control, anaphase and telophase figures were normal. Time-lapse imaging confirmed that anaphase was abnormal after SUMT expression (Fig 7C). While chromosomes were separated apart efficiently during control mitoses in the anterior compartment, an extended metaphase that was followed by an abnormal slow anaphase occurred in the posterior compartment.

For comparison, we performed analogous analyses after expression of SUM (UNO-mCherry) and TEF-EGFP (S7 Fig). SUM resulted in similar mitotic defects as SUMT, although they were milder. In contrast, we did not observe mitotic abnormalities after TEF-EGFP expression.

We conclude that expression of SUM and even more so of SUMT in wing imaginal disc cells is sufficient for establishing conjunction between chromosomes that interferes with their regular segregation during mitosis.

## Discussion

Genes required specifically for alternative homolog conjunction (AHC) during the achiasmate meiosis of *Drosophila* males were identified initially by extensive screening of mutants, and the *teflon* (*tef*) mutant phenotype was the first to be characterized in detail [8]. The molecular identification of the affected gene [9] revealed that *tef* encodes a zinc finger protein. Here, we report a more detailed functional characterization of the TEF protein. Expression pattern and intracellular localization during spermatogenesis were clarified with the help of tagged functional variants. An interaction between TEF and MNM was demonstrated by co-immunoprecipitation, and the responsible binding regions were mapped. Moreover, TEF was shown to bind to chromatin of polytene chromosomes in larval salivary glands. Importantly, TEF recruits MNM to chromatin, and via MNM also the other known AHC proteins SNM and UNO. Moreover, TEF potentiates the chromosome-linking activity of the AHC proteins SNM, UNO and MNM, as revealed by overexpression experiments in spermatocytes and other cell types.

The TEF expression pattern was characterized with *g-tef-sm_myc*. This transgene under control of the *tef* regulatory region results in expression of a TEF version tagged with a spaghetti monster myc epitope tag (sm_myc) [23]. According to mutant rescue experiments, TEF-sm_myc is fully functional. Based on the *g-tef-sm_myc* expression pattern revealed by anti-myc immunofluorescence, TEF is absent or low in somatic hub cells of testes but present in germline stem cells, spermatogonial cells and spermatocytes. TEF-sm_myc is also abundant in ovaries, where it is not germline-restricted as in testes. TEF’s role in ovaries remains unclear, as no aberrant phenotype has been found in *tef* mutants so far [8,9].

The subcellular localization of TEF-sm_myc was unexpected. During spermatogonial mitoses, a strong enrichment on centrosomes was observed. We note that mitotic centrosomal localization is also characteristic of CP190, an architectural chromatin protein, which like TEF has zinc fingers and interacts with a Mod(mdg4) protein [35]. During interphase, TEF-sm_myc was primarily in many intranuclear foci of variable size, and a majority of these did not appear to be chromatin-associated. Intriguingly, the presence of TEF-sm_myc in spermatocytes was transient. TEF-sm_myc levels declined during spermatocyte maturation. It was no longer detectable in late spermatocytes (stages S5 and S6) and during the meiotic divisions. This subcellular localization and transient presence in spermatocytes were also observed in case of *bamP-GAL4-VP16* driven *UASt-tef-EGFP* expression, which also rescues *tef* mutants. Importantly, in contrast to TEF, the other known AHC proteins (SNM, MNM and UNO, abbreviated as SUM) are all detectable on autosomal bivalents, when expressed analogously (as EGFP fusions from *UASt* transgenes with *bamP-GAL4-VP16*) [11,24]. These results argue strongly against the notion that homologous autosomes are conjoined by complexes of AHC proteins containing stoichiometric amounts of TEF. Rather than being an essential component of the glue that keeps homologous autosomes linked until onset of anaphase I, TEF might function only in early spermatocytes in the regulation of AHC establishment. We note that a presence of functional TEF in bivalents of late spermatocytes at levels below detectability is not excluded.

Our comparison of the mutant phenotypes caused by loss of *tef*, on the one hand, and loss of *snm* or *mnm*, on the other hand, provides further arguments against the notion that TEF is an essential component of the glue that conjoins autosomal homologs. The two *tef* alleles present in the transheterozygous mutants that we have analyzed are early non-sense mutations, shown to be amorphic with respect to meiotic chromosome transmission [9]. However, the extent of autosome missegregation was significantly less severe in the transheterozygous *tef* mutants compared to *snm* and *mnm* mutants. This conclusion rests on concurrent findings made by time-lapse imaging of progression through M I and by analyses of meiotic chromosome missegregation with dodeca FISH. In *snm* and *mnm* mutants, bivalents are prematurely separated into independent univalents that are segregated randomly during M I. In contrast, in *tef* mutants there is some residual conjunction of autosomal homologs and their segregation is not completely random. Our phenotypic comparisons are therefore consistent with the notion that TEF contributes to AHC establishment in early spermatocytes rather than also to the maintenance of AHC until anaphase I like the SUM proteiAccording to our observations after ectopic expression of AHC proteins, TEF might contribute to AHC establishment by promoting the recruitment of the SUM proteins to chromatin. TEF is the only AHC protein with a predicted *bona fide* DNA-binding domain. TEF has three zinc fingers, one in the N-terminal and two in the C-terminal region. Jointly, these N- and C-terminal zinc fingers mediate efficient TEF binding of TEF to polytene chromosomes after ectopic expression in larval salivary glands, as deletion of either the N- or the C-terminal region resulted in a substantial reduction of the chromosome-associated signals. Consistent with the absence of known DNA-binding motifs, none of the other AHC proteins displayed substantial binding to polytene chromosomes when expressed individually. Unexpectedly, however, polytene chromosome binding was clearly observed after co-expression of SNM and UNO. Presumably, these two proteins form a complex (SU) that includes a composite DNA-binding site. The two chromosome-binding entities among the AHC proteins, TEF and SU, have distinct preferences for chromosomal locations. However, both are able to recruit MNM onto polytene chromosomes. TEF and MNM interact directly according to our co-immunoprecipitation experiments after transient expression in S2R+ cells, consistent with the previously observed co-purification of TEF with MNM-EGFP from testis extracts [11]. The TEF-MNM interaction is mediated by the N-terminal part of TEF that includes the first zinc finger and by the C-terminal part of MNM. This C-terminal part is uniquely present in MNM. All the many additional isoforms that are generated by differential splicing from the complex *mod(mdg4)* locus have distinct C-terminal parts, and the three isoforms tested (T, C and P) were unable to bind to TEF. Beyond the TEF-MNM interaction, our analyses of polytene chromosome binding and of co-immunoprecipitation suggested that all four AHC proteins can co-assemble into SUMT complexes.

Clearly, in salivary glands, TEF does not just bind to autosomes but also to the X chromosome. Thus, TEF does not appear to have an autosome-specific chromosome-binding ability that would explain why *tef* is required in spermatocytes for regular M I segregation of autosomes but not of sex chromosomes [8]. We would like to suggest that the chromosome-specificity of the *tef* requirement might be linked to an additional effect of TEF on AHC proteins. According to the quantification of expression levels after *Sgs3-GAL4*-mediated expression of AHC proteins in salivary glands, formation of AHC protein complexes appears to stabilize these proteins. Levels of TEF and MNM were higher after co-expression compared to individual expression. Analogous observations were made with SNM and UNO. Similarly, after *bam-GAL4-VP16*-driven overexpression of SUM or SUMT in spermatocytes, the levels of the only tagged protein UNO-mCherry were increased by the presence of TEF. Moreover, MNM-EGFP levels were lower in *tef* mutant spermatocytes. Overall, these observations indicate a positive correlation between TEF and SUM protein levels. In *tef* mutants, some of the remaining SUM is presumably still recruited to autosomal bivalents due to the chromosome-binding activity of SU. However, as SUM levels during wild-type meiosis are far lower on autosomal bivalents compared to the chrXY bivalent, autosomal bivalents might be more strongly affected when SUM protein levels decrease as a result of a loss of *tef* function. TEF increases SUM protein levels presumably by promoting the formation of protein associations that are more stable than the individual proteins. Stimulating effects of TEF on SUM gene transcription are not excluded but unlikely as our analyses included experiments where the AHC proteins were expressed with exogenous regulatory sequences (UAS_GAL4_ and *hsp70*).

Our proposed explanation for the autosome-specific effect of *tef* mutations remains speculative, also because of the technical difficulties to detect SUM proteins on autosomal bivalents. Even the normal amounts of autosomal SUM proteins during wild-type meiosis are difficult to detect unequivocally and consistently in each spermatocyte [10,11]. Here, by analyzing fluorescent versions of UNO, we provide a quantitative estimate for the striking difference in the amount of SUM proteins on autosomes and sex chromosomes in normal spermatocytes. We find around 25-100-fold lower amounts of UNO on autosomal bivalents compared to the chrXY bivalents. Without future technical improvements of detection sensitivity, a conclusive demonstration of the postulated residual autosomal SUM complexes in *tef* mutants is not feasible.

If detectable, the autosomal SUM proteins appear to be confined to 1–2 dots per bivalent at NEBD I in normal spermatocytes. Do these dots mark the location of autosomal homolog conjunction, or might there be additional SUM complexes at other locations below the limit of detection that contribute to conjunction as well? Recent cytological analyses of meiotic quadrivalents in spermatocytes heterozygous for autosomal translocations have indicated that autosomal homolog conjunction is spatially constrained to dot-like chromosomal locations [36]. The spatial control of autosomal homolog conjunction in spermatocytes appears to be analogous to that of canonical crossovers. As a rule, a single restricted region within the euchromatic portion of each autosomal chromosome arm is linked by AHC protein assemblies to its homologous region [36]. Thus, AHC positions might be controlled by processes analogous to crossover interference. The particularly strong crossover interference in *C*. *elegans* has recently been proposed to involve spatially restricted biomolecular condensation of recombination nodule proteins in combination with a regulated coarsening process [37]. It is tempting, therefore, to speculate about the significance of MNM’s apparent liquid phase separation potential. Both MNM and TEF include substantial portions that are predicted to be intrinsically disordered. Such regions are thought to favor liquid-liquid unmixing when they confer multivalent interactions [29]. At high levels of expression, MNM-EGFP formed droplets in salivary gland nuclei, while this was hardly observed with MNM-mCherry. The known weak dimerization of EGFP might reinforce multivalent associations. With TEF-EGFP we did not obtain droplets when expressed alone but when co-expressed with MNM-mCherry, which was co-localized with TEF-EGFP in the droplets. Thus, droplet formation was stimulated by the EGFP tag when present on either MNM or its binding partner TEF. In case of untagged endogenous proteins, droplet formation might remain restricted to chromosomally recruited AHC assemblies. Accordingly, liquid phase separation of AHC proteins might be involved in the control of establishment or maintenance of alternative homolog conjunction in *Drosophila* spermatocytes.

Our experiments with spermatocytes revealed that an excess of AHC proteins is detrimental to regular chromosome segregation during male meiosis. Overexpression of SUMT severely inhibited chromosome territory formation most likely because it results in increased and more widespread conjunction between not only homologous but also non-homologous chromosomes. As a consequence, presumably, chromosomes fail to separate normally, often forming prominent bridges during anaphase and telophase of M I. The meiotic defects observed after SUMT overexpression are highly reminiscent of those caused by a loss of condensin II function [7]. Conversely, absence of SUMT, as in mutants, has very similar phenotypic consequences as overexpression of the limiting condensin II subunit Cap-H2 [7]. Evidently, AHC proteins and condensin II have opposing activities that need to be in proper balance.

The detrimental effects on meiotic chromosome segregation were much stronger after overexpression of SUMT compared to SUM. Overexpression of individual AHC proteins had barely any effect. These results provide further support for our proposal that in normal male meiosis, TEF assists in the chromosomal recruitment of SUM, the actual glue that maintains homolog linkage in bivalents until anaphase I onset. Accordingly, overexpression of TEF alone might not have severe detrimental effects because potentially low levels of endogenous SUM proteins might not allow excess assembly. Similarly, previous overexpression of individual SUM proteins was not observed to cause severe detrimental effects [11,24], perhaps also because low levels of other SUM subunits might limit excess assembly. Our finding that ectopic SUMT expression in mitotically proliferating wing imaginal disc cells results in mitotic defects that resemble closely to the meiotic defects observed after SUMT overexpression in spermatocytes might indicate that AHC during male meiosis does not depend on additional spermatocyte-specific proteins beyond the known AHC proteins. The three proteins SNM, UNO, and MNM appear to be sufficient to induce conjunction between mitotic chromosomes and thus interfere with their normal segregation during anaphase. In combination with TEF, SUM had even more detrimental effects on mitotic chromosome segregation. However, much remains to be learned about the regulation that controls the appropriate chromosomal positioning of AHC during male meiosis.

## Materials and methods

### *Drosophila* lines

Most lines with mutations or transgenes that we have used for our analyses have been described earlier: *tef*^z2-3455^, *tef*^z2-4169^, *UAS-tef* [8,9]; *mnm*^*z3-3298*^, *mnm*^*z3-5578*^, *snm*^*z3-0317*^, *snm*^*z3-2138*^ and *hs-mnm-EGFP* [10]; *UASt-mnm*, *UASt-mnm-EGFP*, *UASt-snm*, *UASt-snm-EGFP* [24]; *uno*^*cc1*^, *g-uno-EGFP* and *UASt-uno-EGFP* [11]; *g-cid-EGFP-cid* and *g-His2Av-mRFP* [38]; *gi2xtdTomato-Cenp-C* [39]; *g-His2Av-GFP* (Bloomington Drosophila Stock Center (BDSC) #5941), *UASt-nls-GFP* (BDSC #4776), *UASt-mCherry-nls* (BDSC #38424), *bamP-GAL4-VP16* [40], *Sgs3-GAL4* (BDSC #6870), *matα4-GAL4-VP16* (BDSC #7062), *GMR-GAL4* (BDSC# 1104), *ey-GAL4* 5–8 [41], *sev-GAL4* [42], *Act5C-GAL4* (BDSC #3954), *en-GAL4* [43], and *αTub84B-GAL80*^*ts*^ (BDSC #7108). For control experiments, we used *w* or *w*^*1118*^ flies unless specified differently.

Fly lines carrying the transgenes *g-tef-sm_myc*, *UASt-tef-myc*, *UASt-tef-EGFP*, *UASt-tef_N-EGFP*, *UASt-tef_C-EGFP*, *UASt-mnm-mCherry*, *UASt-snm-mCherry*, *UASt-uno-mCherry*, *UASt-mCherry-uno-EGFP*, *UASt-mod(mdg4)_CP-mCherry*, *UASt-mod(mdg4)_T-mCherry UASt-mod(mdg4)_P-mCherry UASt-mod(mdg4)_C-mCherry* were generated with the plasmids described below. The pCaSpeR4 and pUASt constructs were injected (BestGene Inc., Chino Hills, CA, USA) into *w*^*1118*^ embryos, the pUASt_attB-mCherry-uno-EGFP construct into *y*^*1*^ *w*^*67c23*^*; P{CaryP}attP2*.

Standard crossing and generation of recombinant chromosomes were used for the production of the various strains used for experimental analyses. The genotypes of the flies analyzed are described in detail in the supporting information (S2 Table). All flies analyzed were raised at 25°C.

While verifying the presence of the *tef* mutations in the lines that we used for analysis, our DNA sequencing of fragments amplified from genomic DNA did not just confirm the expected mutations but also revealed that the *tef* gene on second chromosome balancers appears to include a mutation that is predicted to interfere with regular removal of the first intron during splicing. Inspection of genome re-sequencing data (Bioproject accession numbers PRJNA413446 and PRJNA315473) confirmed the presence of this mutation (2R:17056299 C -> T) in the splice acceptor consensus motif at the end of intron 1, which might cause a severe or even complete loss of *tef* function, on the balancer chromosomes *CyO*, *SM5* and *SM6A* analyzed by [44]. While this finding does not affect any of our conclusions, it is reported for consideration in future analyses.

### Plasmids

A pCaSpeR4 construct was made for the generation *g-tef-sm_myc* transgenic flies. In a first step, a genomic *tef* 3’ region was amplified enzymatically from *w* genomic DNA with the primer pair LV002/LV003 (see S3 Table for oligonucleotide sequences). After digestion of the resulting fragment with KpnI and NotI, it was inserted into the corresponding sites of pCaSpeR4, yielding intermediate 1. The *tef* coding region was also amplified from *w* genomic DNA with LV004/LV005. After digestion of the resulting fragment with NotI and BamHI, it was inserted into the corresponding sites of intermediate 1, yielding intermediate 2. In case of the *tef* 5’ region, amplification was achieved with LV001/LV006 again from *w* genomic DNA. After digestion of the resulting fragment with BamHI and XhoI, it was inserted into the corresponding sites of intermediate 2, resulting in intermediate 3. The construction was completed by amplification of the region coding for spaghetti monster-myc from pCAG_smFP Myc (Addgene plasmid #59757) [23] with LV007/LV008, followed by insertion into the NotI site of intermediate 3.

A pUASt construct was made for the generation *UASt-tef-10xmyc* transgenic flies. A region coding for the myc epitopes was excised from pCaSpeR4-gMis12-10xmyc [45] using BamHI and XbaI. The resulting 484 bp fragment was inserted into the BglII and XbaI sites of pUASt to create the first intermediate. This intermediate was opened with EcoRI to insert a double-stranded oligonucleotide obtained by annealing LV026 and LV027, which destroyed the EcoRI site and introduced a NotI restriction site. This NotI site was used for the insertion of the *tef* coding sequence which was amplified from the EST plasmid LD32631 using AB140/AB141.

For the generation *UASt-tef-EGFP* transgenic flies, we also generated a pUASt construct. The *tef* coding region was amplified from pUASt-tef-10xmyc using OL005/CL337. After digestion of the resulting fragment with NotI and XhoI, it was inserted into the corresponding sites of pUASt-mcs-EGFP [45]. Derivatives of the resulting pUASt-tef-EGFP construct were used for the generation of *UASt-tef_N-EGFP* and *UASt-tef_C-EGFP* transgenic fly lines. For the former, we replaced the SpeI-XhoI fragment in pUASt-tef-EGFP with a double-stranded oligonucleotide obtained by annealing ZK048/ZK049. In case of the latter, the NotI-SpeI fragment in pUASt-tef-EGFP was replaced with the double-stranded oligonucleotide obtained by annealing ZK050/ZK051. The plasmid UASt-tef_N-EGFP codes for amino acid residues 1–292 of TEF and pUASt-tef_C-EGFP codes for a TEF variant with amino acid residues 1–3 followed by 287–649.

For the construction of pUASt-mnm-mCherry, we digested pUASt-mnm-EGFP [24] with NotI to release the *mnm* coding region, which was then inserted into the corresponding site of pUASt-mcs-mCherry. Analogously, pUASt-snm-mCherry was made using pUASt-snm-EGFP [24]. For the construction of pUASt-uno-mCherry, the *uno* coding sequence was isolated from pUASt-uno-EGFP [11] by digestion with EcoRI and XhoI, followed by transfer into the corresponding sites of pUASt-mcs-mCherry. This latter vector was generated by insertion of the mCherry coding region into the XhoI and XbaI sites of pUASt after amplification of the DNA insert fragment with CL302 and PVL075 followed by digestion with XhoI and XbaI.

For the production of *UASt-mCherry-uno-EGFP* transgenic lines, we made a pUASt-attB derivative [46]. After amplification of the EGFP coding region with RAS079/RAS080, it was digested with KpnI and XbaI and inserted into the corresponding sites of pUASt_attB, yielding pUASt_attB-mcs-EGFP. After amplification of the mCherry coding region with PVL042/OL004 and digestion with EcoRI and BglII, it was added into the corresponding sites of pUASt_attB-mcs-EGFP, yielding intermediate 2. Finally, the uno coding region was amplified from pCaSpeR5-guno-EGFP [11] with JW122/JW123, digested with BglII and XhoI and inserted into the corresponding sites of intermediate 2.

For the generation of pUASt-mod(mdg4)_CP-mCherry, pUASt-mod(mdg4)_T-mCherry, pUASt-mod(mdg4)_C-mCherry and pUASt-mod(mdg4)_P-mCherry, we replaced the XhoI-KpnI fragment from pUASt-mnm-mCherry with the synthetic DNA fragments CL345 –CL348.

Many of the pUASt constructs described above (pUASt-tef-10xmyc, pUASt-tef-EGFP, pUASt-tef_N-EGFP, pUASt-tef_C-EGFP, pUASt-mod(mdg4)_CP-mCherry, pUASt-mod(mdg4)_T-mCherry, pUASt-mod(mdg4)_C-mCherry, pUASt-mod(mdg4)_P-mCherry, pUASt-mnm-EGFP, pUASt-mnm-mCherry, pUASt-snm-EGFP, pUASt-snm-mCherry, pUASt-uno-EGFP, pUASt-uno-mCherry) were also used for transient expression in S2R+ cells after co-transfection with pCaSper4-Act5C-GAL4 (kindly provided by Christian Klämbt, Westfälische Wilhelms-Universität, Germany). In addition, pUASt-mnm [24] and pUASt-nls-tetR-EGFP (kindly provided by Stefan Heidmann, Universität Bayreuth, Bayreuth, Germany) were used for transient expression in S2R+ cells. Moreover, we generated additional pUASt constructs for transient expression in S2R+ cells. For the construction of pUASt-tef-mCherry, we transferred the XhoI-XbaI fragment from pUASt-tef-EGFP into the corresponding sites of pUASt-mcs-mCherry. The plasmids pUASt-tef_Na-EGFP, pUASt-tef_Nb-EGFP, pUASt-tef_Nc-EGFP, and pUASt-tef_Nd-EGFP were generated with the synthetic DNA fragments CL398 –CL401 replacing the NotI-XhoI fragment in pUASt-tef_N-EGFP.

### Cell culture and transfection

S2R+ cells were cultured in Schneider’s medium (Gibco, cat# 21720, Thermo Fisher Scientific, Waltham, MA), 10% fetal bovine serum (Gibco, cat# 10500–064) and 1% Penicillin-Streptomycin (Gibco, cat# 15140) at 25°C. Transfections were performed using FuGENE HD (Promega, cat# E2311) in T25 or T75 flasks. In T25 flasks, 5.2×10^6^ cells were plated in 4 ml complete medium, in T75 flasks, 15.6×10^6^ cells in 8 ml complete medium. One hour after plating transfection mix was added. In case of T25 flasks, 200 μl of transfection mix was used containing 2 μg plasmid DNA and 8 μl FuGENE HD in Schneider’s medium. For T75 flasks, 400 μl of transfection mix was used containing 4 μg plasmid DNA and 16 μl FuGENE HD in Schneider’s medium. Cells were incubated for 2 days before analysis in co-immunoprecipitation experiments.

### Immunoprecipitation and immunoblotting

S2R+ cells were harvested with a cell scraper and the resulting cell suspension was centrifuged at 580 x g for 5 minutes in a 15 ml Falcon tube. The cell pellet was washed with 1 ml cold phosphate-buffered saline (PBS) (137 mM NaCl, 2.7 mM KCl, 1.47 mM KH_2_PO_4_, 6.46 mM Na_2_HPO_4_, pH 7.4) and transferred to a 1.5 ml Protein LoBind tube (Eppendorf, cat# 022431081), which were also used for all subsequent steps. Cells were sedimented at 600 x g for 5 minutes at 4°C. All subsequent steps were performed on ice or at 4°C with ice cold solutions. Cells were lysed in lysis buffer (LB): 20 mM Tris-HCl pH 7.5, 300 mM NaCl, 2 mM MgCl_2_, 0.1% Nonidet P-40 Substitute (Sigma Aldrich, cat# 74385), 5% glycerol, 0.5 mM EGTA, 1 mM DTT, 50 U/ml Benzonase Nuclease ultrapure (Sigma Aldrich, cat# E8263) and 1 tablet Roche protease inhibitor c0mplete per 10 ml lysis buffer (Mini EDTA-free, EASYpack, Roche, cat# 04693159001). The cells harvested from a T25 flask were lysed in 500 μl LB and in 1000 μl LB in case of T75 flasks by pipetting up and down twice, each time followed by a 15-minute incubation. Cell lysates were cleared by centrifugation for 15 minutes at 16100 x g. A small aliquot of the supernatant was removed for analysis by immunoblotting (input samples). The rest of the supernatant was added to pre-washed 25 μl nano-trap agarose beads (ChromoTek, GFP-Trap agarose, cat# gta-20, or RFP-Trap agarose, cat# rta-20). Beads were incubated for 1 hour on a rotating wheel at 15 rpm. Thereafter, beads were washed 3 times with LB and centrifugation at 2500 x g for 2 minutes. For the final wash samples were transferred into a fresh tube. For elution, beads were resuspended in 80 μl 3x Lämmli Buffer (62.5 mM Tris-HCl, 10% glycerol, 5% β-mercaptoethanol, 3% SDS, 0.01% Bromophenol Blue) and boiled for 8 minutes at 96°C. After rapid cooling on ice, beads were sedimented and the supernatant was distributed in three aliquots of 25 μl (IP samples). All samples were snap frozen in liquid nitrogen and stored at -80°C until analysis by immunoblotting.

Samples were resolved by sodium dodecyl sulfate-polyacrylamide gel electrophoresis (SDS-PAGE) using a mini-gel system (BioRAD) and gels with between 7% and 12% polyacrylamide for 3 hours at 90 V. Molecular weight markers were PageRuler Plus Prestained Protein Ladder (Thermo Scientific, cat# 815-968-0747). Protein transfer to nitrocellulose membranes (Amersham Protran 0.45 μm, cat# 10600002) was achieved by tank blotting at room temperature for 1 hour with 100 V. After transfer, Ponceau S staining was performed, followed by a blocking step using 5% dry milk (w/v) in PBS with 0.02% NaN_3_. This solution was also used for incubation with the primary antibody for 2 hours at room temperature or overnight at 4°C. Three washes with 5% dry milk (w/v) in PBS were done before incubation with the secondary antibody, which was applied in 5% dry milk (w/v) in PBS at room temperature for 1 hour protected from light. After 2 washes with 5% dry milk (w/v) in PBS and 2 washes with PBS, 0.1% Tween-20, signals were detected with ECL reagents (WesternBright ECL, Advansta, cat# K-12045-D50) in an Amersham Imager 600.

The following antibodies were used for immunoblotting: rabbit polyclonal antibodies anti-GFP diluted 1:800 (ChromoTek, cat# pabg1) or 1:2000 (Torrey Pines Biolabs, cat# TP401); mouse monoclonal antibody anti-RFP (ChromoTek, cat# 6g6), 1:1000; rat monoclonal antibody anti-c-MYC (ChromoTek, cat# 9e1) at 1:1000; rabbit polyclonal antibody anti-ModC [13] (kindly provided by Rainer Dorn, Universität Halle, Halle, Germany) at 1:4000; HRP-conjugated AffiniPure goat anti-rabbit IgG polyclonal antibody (Jackson ImmunoResearch, cat# 111-035-003) at 1:1000; HRP-conjugated AffiniPure goat anti-mouse IgG polyclonal antibody (Jackson ImmunoResearch, cat# 115-035-003) at 1:1000; HRP-conjugated goat anti-rat IgG antibody (Thermo Scientific, cat# 62–9520) at 1:5000.

### Ectopic AHC protein expression during development and analysis of associated toxicity

For ectopic expression of AHC proteins during development, crosses were set up with a single male providing a particular *GAL4* driver transgene (*ey*-, *sev*-, *GMR*-, *Act5C*-, *αTub84B*- or *en-GAL4*) and a single female contributing one or several distinct *UASt* transgenes to the F1 progeny. In case of the control crosses, the female did not contribute any *UASt* transgene. Five parallel crosses were set up for each genotype (see S1 Table) using flies aged up to 2 days after eclosion. These flies were homozygous for the transgenes, except in case of *Act5C*- and *αTub84B*-GAL4, where balanced males were used. The crossed flies were transferred into fresh vials twice after allowing egg deposition for 4–5 days in between. After development at 25°C, all the F1 progeny eventually eclosing in the three vials resulting from each cross were counted. In cases of premature death of a parent before all three vials had been started, the cross was discounted and replaced with a fresh cross. The total number of progeny obtained from the control crosses was set as 100%, except in the crosses with the balanced *Act5C*- and *αTub84B*-GAL4 males. In these crosses, the total number of balanced progeny was set as 100%. To document the eye phenotypes of adult F1 progeny flies, their head region was imaged with a stereomicroscope (Leica MZ16F) and a color camera (Zeiss AxioCam HRc). Around 15 focal planes spaced manually by about 15 μm were acquired, followed by focus stacking with Helicon software.

### Analysis of larval salivary glands after ectopic AHC protein expression

The *Sgs3-GAL4* driver and *UASt* transgenes coding for AHC proteins with and without fluorescent protein tags were crossed together, and wandering third instar larvae were used for dissection of salivary glands after development at 25°C. Salivary gland preparations were made similar as previously described [47,48]. After a brief rinse in water, larvae were transferred for dissection into freshly prepared formaldehyde fixation buffer (FBA) (0.15 M piperazine-N,N′-bis(2-ethanesulfonic acid), 3 mM MgSO_4_, 1.5 mM ethylenediaminetetraacetic acid, 1.5% IGEPAL CA-630, pH 6.8). After three minutes for dissection and fixation, glands were transferred to phosphate-buffered saline (PBS) containing 0.1% (wt/vol) Triton X-100. Moreover, the solution also contained the DNA stain Hoechst 33528 at a concentration of 2 μg/ml for whole-mount preparations and at 5 μg/ml for squash preparations. After an incubation of five minutes, glands were washed with 50% (wt/vol) glycerol for five minutes and then transferred into an 18-μl drop of 50% glycerol on a slide. A coverslip was gently lowered onto the glands. In case of squash preparations, release of polytene chromosomes from the nuclei was promoted by tapping onto the coverslip with forceps. Preparations were sealed with nail polish.

Polytene chromosomes in squash preparations were imaged by acquiring single focal planes with a wide-field fluorescence microscope (Zeiss Axio Observer HS) and a Plan-Aprochromat 100x/1.4 objective. Whole-mount preparations were used for an analysis of expression levels by microscopic quantification of signal intensities. Images were acquired with an EC Plan Neofluar 10x/0.3 objective from the distal part of the gland that contained the basal cells. Fluorescent signals in the green, red and blue channels within a single optical section were acquired at the z position, where a maximal number of nuclei were in focus. Fiji in combination with a macro was used for the quantification of signal intensities. Briefly, a region of interest (ROI) was drawn manually to include all of the salivary gland tissue present in the microscopic image, but not any adherent residual fat body parts or other regions outside the salivary gland. Within the manually selected salivary gland ROI, the salivary gland nuclei were identified automatically based on the signals of the DNA staining. After applying a Gaussian blur, the plugin “Auto Local Threshold” was used to define the nuclei as objects, followed by separation of touching nuclei using the Image J watershed method and filtering out objects with a circularity and a size outside an appropriate range. Finally, the mean pixel intensities within the nuclear ROIs, as well as outside of these ROIs (i.e., in the cytoplasm), were quantified in the red and the green channel. Values obtained from 3–8 distinct glands per genotype were averaged.

### Analysis of embryos after ectopic AHC protein expression

Embryos were collected from a cross of w*; *mat-GAL4-VP16*/*CyO*, *P{ry[+t7*.*2] = ftz-lacB}E3* virgins and males with *UASt* transgenes coding for AHC proteins. Egg collection was done at 25°C on apple agar plates for three hours, followed by ageing for three additional hours. Embryos were fixed with 4% formaldehyde as described [49]. For DNA staining, the fixed embryos were incubated for 5 min 1 μg/ml Hoechst 33285 in PBST (PBS containing 0.1% Triton X-100). After three washes with PBS, embryos were mounted in mounting medium (70% (wt/vol) glycerol, 50 mM Tris·HCl, pH 8.5). Image stacks (7 z sections with 600 nm spacing) were acquired from embryo regions with cells in the G2- or M phase of cycle 14 or in early interphase of cycle 15 with a wide-field fluorescence microscope (Zeiss Axio Observer HS) and a Plan-Apochromat 100x/1.4 objective. Maximum intensity projections are displayed (S6 Fig).

### Analysis of wing imaginal discs after ectopic AHC protein expression

Eggs were collected from a cross of w*; en-GAL4, *αTub84B-GAL80*^*ts*^*/ CyO*, *P{Dfd-EYFP}2* virgin females and males with *UASt* transgenes coding for AHC proteins for 40 hours at 18°C in a bottle with standard fly food. After seven additional days at 18°C, the bottle was shifted to 29°C for 16 hours, followed by dissection of wing imaginal discs from third instar wandering stage larvae at room temperature in complete S2R+ cell culture medium. For live imaging, wing discs were transferred into S2R+ cell culture medium containing the fluorescent live DNA stain SPY555-DNA (Spirochrome AG, Stein am Rhein, Switzerland, #sc201) at a dilution of 1:2000. Imaginal discs were covered with an agarose slab (1% in complete Schneider’s medium). Image stacks (40 z-sections with 500 nm spacing) were acquired at 1 min intervals at 25°C. Movies or still frames were exported as maximum intensity projections using IMARIS software with interpolated image display. For analysis of fixed wing imaginal discs, we transferred the dissected imaginal discs for fixation into 4% PFA in PBS for 20 minutes. After three washes with PBST each for 5 minutes, DNA staining was performed for 10 min in PBST containing 1 μg/ml Hoechst 33285. After three washes with PBS each for 5 minutes, imaginal discs were mounted between a slide and a coverslip in Vectashield Antifade Mounting Medium (Vector Laboratories, cat# H-1000) with the peripodial membranes facing the coverslip. A wide-field fluorescence microscope (Zeiss Axio Observer HS) with a Plan-Aprochromat 100x/1.4 objective was used for acquisition of image stacks (about 20 z-sections with 500 nm spacing) covering the region between the peripodial membrane and the disc epithelium, which contains the mitotic cells. Maximum intensity projections with primarily the mitotic cells (about 5 z-sections) are displayed (Figs 7B and S7).

### Fixation and labeling of ovary and testis preparations

For whole-mount testis preparations, dissections from young adult males (0–1 day after eclosion) were performed in testis buffer (183 mM KCl, 47 mM NaCl, 10 mM Tris-HCl, pH 6.8). Testes were fixed in PBST containing 4% formaldehyde in 0.2 ml Eppendorf tubes for 20 minutes on a rotating wheel. For whole-mount preparations of ovaries, these organs were dissected from adult females in PBS. Ovarioles were gently teased apart before fixation in 4% formaldehyde for 15 minutes on a rotating wheel. Testis squash preparations were made and stained essentially as described previously [50]. For immunolabeling, mouse monoclonal antibody 9B11 anti-myc (Cell Signaling Technology, #2276) was used at 1:1000. Secondary antibodies were Alexa488-conjugated goat antibodies against mouse IgG (Invitrogen, A11004) diluted 1:500 in PBST containing 5% fetal bovine serum. Washing after fixation and after incubation with primary and secondary antibodies was performed with three incubations in PBST for 15 min each. For DNA staining, testes were incubated for 10 minutes in PBS, 0.1% Triton X-100 (PBTx) containing Hoechst 33258 (1 μg/ml). After three washes with PBS, preparations were mounted under a coverslip in a drop of mounting medium.

For FISH labeling, testes were dissected in testis buffer and transferred into a drop of testis buffer on a poly-L-lysine treated slide. Testes were torn open with forceps and gently squashed under a siliconized coverslip to release cysts. Preparations were frozen immediately in liquid nitrogen. Thereafter, the coverslip was flipped off and slides were immersed into ethanol at -20°C for 10 min before additional fixation with 4% formaldehyde in PBS. For permeabilization, slides were incubated in PBS containing 0.3% Triton X-100 and 0.3% sodium deoxycholate for 15 min twice. Slides were washed four times in PBST. Ethanol incubations and dehydration with a formamide series were done as described (immuno-FISH protocol 3.2, steps 10–26) [51]. An oligonucleotide (5’-CCCGTACTGGTCCCGTACTGGTCCCGTACTCGGTCCCGTACTCGGT-3’) with Atto-565 on both the 5’- and the 3’ end (Integrated DNA Technologies, Leuven, Belgium) was used for detection of the chr3-specific dodeca satellite sequences at a concentration of 1 ng/μl in hybridization buffer. The denaturation step was performed at 98°C for 6 min, and hybridization over night at 37°C. Slides were washed twice for ten minutes for each wash with 50% formamide, 2x SSCT at 37°C. Thereafter, additional washes of ten minutes were performed at room temperature, first once in 25% formamide, 2x SSCT and then three times in 2x SSCT. DNA was stained with Hoechst 33258 (1 μg/ml) for 10 minutes. Before mounting, slides were washed twice in PBS for 5 minutes.

For determination of the rate of chr3 missegregation during the male meiotic divisions, the dodeca FISH assay has some limitations. Apart from irregular segregation during M I and/or M II, chromosome loss and technical problems with FISH signal scoring need to be taken into consideration. While in the control (*w*) the large majority (94%) had a single nuclear dot signal, as expected, nuclei with two dot signals (n2) were counted 32-fold more often than nuclei without a dot (n0). This substantial excess of n2 over n0 in the control is difficult to explain by meiotic chromosome missegregation, pointing to a dot scoring problem. Chr3 harbors two dodeca satellite blocks close to the centromere [52], which might get stretched apart occasionally in some of the spermatid nuclei. Irrespective of potential target region stretching, spermatid nuclei with up to four dots can arise in principle after chr3 missegregation during both meiotic divisions. Spermatid nuclei with more than two dot signals of comparable intensities were observed neither in control nor in the mutants (n > 185 from at least three distinct cysts). However, spatial overlap of dot signals in the maximum intensity projections is possible in principle. Overall, therefore, the number of nuclei without a dot signal might be a more accurate measure of chr3 non-disjunction and loss. We point out that also by taking only the fraction of nuclei with zero dots into account, the extent of chr3 missegregation remains significantly lower in *tef* compared to *mnm* or *snm* mutants.

As an alternative assay for the comparison of chromosome missegregation during the male meiotic divisions in distinct genotypes, microscopic quantification of the DNA content of early spermatid nuclei was applied as described [24].

Preparations of ovaries and testes were analyzed with a wide-field fluorescence microscope (Zeiss Axio Observer HS) using 40×/1.3, 63×/1.4 and 100×/1.4 oil immersion objectives. Maximum intensity projections of image stacks are presented.

For the comparison of nucleolar MNM-EGFP intensities in the presence and absence of *tef* function, testis squash preparations were made using males of the following two genotypes: (1) w*;; *hs-mnm-EGFP*/ *gi2xtdTomato-Cenp-C* and (2) w*; tef^*z2-3455*^/ *tef*^*z2-4169*^; *hs-mnm-EGFP*/ + (see S2 Table for detailed genotype description). Six testes from each genotype were combined for simultaneous squashing under one coverslip, followed by fixation and staining, in order to minimize potential fixation artefacts. Mouse monoclonal antibody ADL67 anti-Lamin Dm0 (Developmental Studies Hybridoma Bank, University of Iowa, Iowa City, USA) (1:40) and Cy5-conjugated goat antibodies against mouse IgG (Invitrogen, A10524) (1:500) were used for immunolabeling/Hoechst 33258 (1 μg/ml) for DNA staining. Regions with spermatocyte cysts at the stages S5 and S6 were identified based on their characteristic DNA staining pattern using a wide-field fluorescence microscope (Zeiss Axio Observer HS) and a Fluar 40x/1.3 oil immersion objective. By acquiring images of the blue and red channels at a single medial z-position, the genotypes (1) or (2) were assigned, because the former but not the latter displayed red centromeric 2xtdTomato-Cenp-C signals. In addition, an image stack with 25 z-sections spaced by 280 nm was acquired from the blue, green and far-red channels. Maximum intensity projections were used for MNM-EGFP signal quantification. A circular ROI with a fixed diameter greater than that of nucleoli was placed manually to include all the MNM-EGFP foci in a given nucleolus. For background subtraction, a slightly dilated ROI was used for the estimation of background intensity around the periphery of the smaller ROI.

### Live imaging with testis preparations

Time-lapse imaging of progression through meiosis was performed as described [20]. Testes were dissected from pupal or young adult males in Schneider’s *Drosophila* Medium (Invitrogen, #21720) supplemented with 10% fetal bovine serum (Invitrogen) and 1% penicillin/streptomycin (Invitrogen, #15140). The dissected pupal testes were transferred into 45 μl of medium in a 35 mm glass bottom dish (MatTek Corporation, #P35G-1.5-14-C) and opened with fine tungsten needles to release the cysts. In case of adult testes, 150 μl of medium were used. To reduce sample movements, 15 μl of 1% w/v methylcellulose (Sigma, #M0387) was added to pupal testes preparations and 50 μl to adult testes preparations. A wet filter paper was placed inside along the dish wall before sealing the lid with parafilm. For live DNA staining, SPY555-DNA (cat# SC201, Stein am Rhein, Switzerland) was added to the preparations at a final dilution of 1:2000.

Imaging was performed at 25°C in a room with temperature control using a spinning disc confocal microscope (VisiScope with a Yokogawa CSU-X1 unit combined with an Olympus IX83 inverted stand and a Photometrics evolve EM 512 EMCCD camera, equipped for red/green dual channel fluorescence observation; Visitron systems, Puchheim, Germany). The data shown in Fig 1 was acquired with a 100×/1.4 oil immersion objective, and the acquired z-stacks comprised 31 focal planes spaced by 500 nm and recorded at 10 seconds intervals. The data shown in Fig 3E was acquired with a 60×/1.42 oil immersion objective, and the acquired z-stacks comprised 33 focal planes spaced by 500 nm and recorded at 10- or 45-second intervals. These parameters were also used in case of the data shown in Fig 6C–6F, except that stacks with 40 z-sections were acquired.

Image processing and analysis in case of the data shown in Fig 1 were done after deconvolution of the image data as described previously [20]. Briefly, kinetochores were identified based on the Cid-EGFP signals and tracked using the spot detection option of the IMARIS software (Oxford Instruments) with the expected radius chosen as 400 nm. The spatial coordinates of the kinetochore positions were exported and used for the calculation of kinetochore velocities (V_KT_) and the distances separating the two kinetochores associated with a given bivalent (D_kt_). The dot formed by MNM-EGFP on the chrXY pairing region was identified and tracked in the same manner as the kinetochores. For quantification of the EGFP signal intensities associated with the kinetochores and the pairing center of the chrXY bivalent, we used the intensity sum observed within the spheres identified by the spot detection option of the IMARIS software without background correction.

For the quantification of signal intensities of fluorescent UNO variants on bivalents around NEBD I, images stacks acquired during time-lapse imaging were opened with IMARIS software followed by changing the voxel dimension from 0.268 x 0.268 x 0.5 to 1 x 1 x 1 μm, which approximately reverses the distortion of the UNO dot signals into ellipsoids elongated along the z-axis by the point spread function of the imaging system. The strong UNO dot signals associated with the chrXY pairing region were identified automatically using the spot detection option of the IMARIS software with the expected radius chosen as 15 μm. The intensity sum of the signals within the resulting spheres was exported for quantification. For subtraction of local background intensities, tracking of the signals associated with the chrXY pairing region was repeated, although with the expected radius increased to 17 μm. The outer region that was contained within the larger sphere (d = 17 μm) but not within the smaller sphere (d = 15 μm) was used for the calculation of an average local background intensity per voxel. The resulting value was then used for background correction of the intensity sum of the signals detected within the smaller sphere. The UNO dot signals associated with autosomal bivalents were quantified analogously. However, the expected radii of the smaller and larger spheres were adjusted to 10 and 12 μm, respectively, and the positioning of the spheres during spot detection with the IMARIS software was done manually. The background-corrected intensity values of the UNO dots either associated with the chrXY bivalent or with the autosomal bivalents that we observed over time during prometaphase I were averaged. The number of time points that were averaged varied from 2 to 25 time points. The variation in the number of averaged time points resulted because the time window for analysis had to be restricted in each cell to the initial period of prometaphase I, during which the weak autosomal UNO dots were still well separated spatially from the far stronger UNO dots on the chrXY bivalent to preclude signal spill-over from the strong into the region with weak dots. In case of the chr4 bivalent, which usually maintains a close association with the sex chromosomes during spermatocyte maturation and early M I [20], the associated UNO dot was sufficiently far away from the UNO dot on the chrXY pairing region only in a subset of cells (see S4 Table) and at relatively few time points. The mean intensity values observed over time were averaged over all analyzed cells of a given genotype.

In case of the analyses presented in Fig 6, the onset of NEBD I was determined as the time point before the diffuse nucleoplasmic His2Av-mRFP or His2Av-EFGP signals started to fade rapidly from the nucleus entering first into M I. We only analyzed cysts that started with NEBD I within 2 hours and 30 minutes after the onset of time-lapse imaging to avoid inclusion of photo-damaged unhealthy cysts. For the analysis of the number of major chromosome territories (Fig 6E), the eleven time points preceding NEBD I were analyzed. For analysis, the 3D reconstruction of the z-stack was rotated using IMARIS software during inspection and the number of completely separated major chromosome territories in all the nuclei of the cyst that were completely contained within the z-stack was determined. Thus, the small chr4 was not considered in this analysis. Two territories that were merging into one for longer than 5 of the 11 analyzed time points were counted as one. The fraction of spermatocytes having one, two or three major territories was calculated with Microsoft Excel and visualized using GraphPad Prism.

For the analysis of chromosome segregation during M I (Fig 6F), cysts were analyzed during exit from M I at around 60 minutes after the onset of NEBD I. For scoring chromosome segregation defects during exit from M I, the 3D reconstructions of the z-stack were again rotated during inspection using IMARIS software. Cells were scored as displaying anaphase bridges (AB), if this revealed transient or permanent His2Av-mRFP positive bridges or also micronuclei between telophase nuclei. If the His2Av-mRFP positive chromosomes were retracted back into one single large nucleus late during M I instead of becoming partitioned onto two daughter nuclei, the cell was scored as affected by a complete separation failure (CSF). If the His2Av-mRFP- or His2Av-GFP-positive chromosomes were partitioned into two round daughter nuclei with strikingly unequal volumes, the cell was counted as displaying asymmetric daughter nuclei (ADN). Finally, if two equally sized round daughter nuclei were generated, the cell was counted as a case with symmetric daughter nuclei (SDN). The fraction of the distinct types of segregation events were calculated with Microsoft Excel and visualized with GraphPad Prism.

For the analysis of UNO-mCherry signal intensities (Fig 6D), we analyzed nuclei completely contained within the z-stack at the time point defined as the onset of NEBD I. The z-stack acquired at this time point was analyzed using ImageJ (1.52i, Java 1.8.0_172, 64-bit) using a macro. A maximum intensity projection of the 40 z-planes was generated. Regions of interest (ROIs) were drawn manually around the intense red dots on the chrXY bivalents. For quantification of the autosomal signals, ROIs were drawn manually around all of the chromosomes using the signals in the His2Av-GFP channel. The chrXY region was included in this ROI, but the chrXY-associated signals were eventually subtracted as described further below. For background correction, the macro generated ROIs enlarged by 2 pixels in case of the chrXY signals and 3 pixels in case of the ROIs around all the chromosomes. The average pixel intensity obtained in the region between the smaller and larger ROIs was used as a measure of background fluorescence, which was subtracted from the total signal intensity observed in the smaller ROI. After background correction, the chrXY signal was subtracted from the total chromosomal signal to yield the autosomal signals. GraphPad Prism was used for visualization of the UNO-mCherry signal intensities on chrXY and autosomes, respectively.

Maximum intensity projections were generated using ImageJ for wide-field images and IMARIS (Bitplane; versions 8.4.0, 9.2.0, 9.7.2) for spinning disk confocal images. Figures display maximum intensity projections unless stated otherwise. Export of projections from IMARIS as movies or still frames after live imaging was made with interpolated image display. Moreover, display parameters for the His2Av-mRFP or His2Av-GFP signals were adjusted manually over time to reveal chromosomes clearly throughout the movies, thereby correcting photobleaching and partially also the changes in the extent of chromosome condensation during M I. Graphs were generated with Microsoft Excel or GraphPad Prism. P values were calculated using a two-tailed student t-test (* = p < 0.05; ** = p < 0.01; *** = p < 0.001). Adobe Photoshop and Adobe Illustrator were used for production of figures.

## Supporting information

S1 Fig Quantification of meiotic chromosome missegregation by DNA content analysis in early postmeiotic spermatid nuclei. (**A**,**B**) DNA signal intensity was quantified for each early spermatid nucleus after imaging squash preparations of testes labeled with a DNA stain. The mean of all the individual nuclear DNA content values obtained for a given cyst was used for normalization of these values, followed by calculation of a normalized standard deviation (nsd). The analyzed genotypes were *w* (+), *mnm*^*z3*-3298^/*mnm*^*z3*-5578^ (*mnm*), *snm*^*z3*-0317^/*snm*^*z3*-2138^ (*snm*), *tef*^z2-3455^/*tef*^z2-4169^ (*tef*), *tef*^z2-3455^/*tef*^z2-4169^; *g-tef-sm_myc* III.1 (*tef*, *g-tef-myc*), *tef*^z2-3455^/*tef*^z2-4169^; *bamP-GAL4-VP16*/*UASt-tef-myc* III.1 (*tef*, *bam> tef-myc*), and *tef*^z2-3455^/*tef*^z2-4169^; *bamP-GAL4-VP16*/*UASt-tef-EGFP* III.2 (*tef*, *bam> tef-EGFP*). (**A**) Bar diagram summarizing DNA content variation. Each green dot represents the nsd of a given cyst. Bars indicate the nsd average across all analyzed cysts with whiskers displaying its standard deviation. DNA content variation in *tef* mutants is significantly different from that in *mnm* and *snm* mutants, and from that in *tef* mutants with the indicated *tef* transgenes (*** p < .001, t-test). (**B**) Plots displaying the normalized DNA content values for all the analyzed nuclei. Values from a given cyst are displayed in the same color, alternating between black and red for different cysts. The cysts analyzed for a given genotype are arranged with increasing nsd from left to right. The nsd average and the number of analyzed cysts are indicated in the upper right boxes.
(PDF)

S2 Fig Expression and subcellular localization of TEF-myc in gonads. (**A**) Ovarioles from *w*^*1118*^ control (+) and *g-tef-sm_myc* transgenic females with either the insertion II.1 or III.1, as indicated, are shown after immunolabeling with anti-myc and a DNA stain. A high magnification of a germarium is shown in the bottom row with arrowheads indicating spindle poles in a mitotic oogonial cyst. (**B**) Whole-mount preparations of testes from *w*^*1118*^ control (+), *g-tef-sm_myc* II.1 and *bam>tef-myc* III.1 males were immunolabeled with anti-myc and a DNA stain. Identical exposure and display settings were used for the control and *g-tef-sm_myc* II.1 images, but an adjusted lower sensitivity was used in case of *bam>tef-myc* III.1 in order to avoid signal saturation in the green channel (anti-myc). A spermatogonial cell in metaphase with anti-myc signals on spindle poles is shown at high magnification (inset). In these whole-mount preparations, the specific anti-myc signals were overall less intense and less granular compared to those in flat testis preparations (Fig 2). (**C**) Germarium and early egg chambers from an ovariole of a *bam>tef-myc* III.1 female after immunolabeling with anti-myc and a DNA stain. Scale bars = 50 μm (A, top three rows), 10 μm (A, bottom row), 50 μm (B) and 20 μm (C).
(PDF)

S3 Fig Level of AHC protein expression in larval salivary glands and MNM droplet formation. (**A,B**) Whole-mount preparations of larval salivary glands stained for DNA were used for comparison of the expression levels of various fluorescently tagged AHC proteins after expression with *UASt* transgenes and *Sgs3-GAL4*. (**A**) Representative images acquired using a 10x objective from the distal part of the gland with the basal cells from *Sgs3>nls-GFP* (top) and *Sgs3>nls-mCherry* (bottom) larvae. Identical exposure times were used for acquisition of a single equatorial focal plane from the green, red and blue channels for all the genotypes. Bar diagram displays mean pixel intensities (a.u.) detected in either the cytoplasm or in the nucleus in the green and red channels for the indicated genotypes (E: EGFP; C: mCherry). The number of analyzed glands (n) is indicated above the genotypes and s.d. by whiskers. The dashed blue line indicates background fluorescence intensity observed in the absence of expression of a fusion with EGFP or mCherry. (**B**) MNM-EGFP droplet formation. The three images on the left side display sub-regions of glands imaged as described above. The three images shown on the right side display images of nuclei acquired with a 100x objective (maximum intensity projections of four optical sections with 500 nm spacing). All images show a merge of the green, red and blue channels, with the DNA staining in the blue channel as grey values. Strong expression of MNM-EGFP results in the formation of intranuclear droplets. In contrast, such droplets are not detected after expression of MNM-mCherry or TEF-EGFP. The latter two fusion proteins are precisely co-localized after co-expression, shifting from chromosome-associated bands into intranuclear droplets (arrowheads in middle panel) with increasing expression levels. Scale bars = 100 μm (A), 50 μm (B, left side) and 10 μm (B, right side).
(PDF)

S4 Fig Expression of Mod(mdg4)-mCherry isoforms in larval salivary glands. *Sgs3-GAL4* and *UASt* transgenes coding for TEF-EGFP and the Mod(mdg4) isoforms CP, T, P and C tagged with mCherry were used for expression specifically in larval salivary glands. Whole-mount preparations were stained for DNA and imaged using identical exposure times. Representative single optical sections of the regions with the distal basal cells of the indicated genotypes are shown (top). Bar diagram displays mean pixel intensities (a.u.) detected in either the cytoplasm or the nucleus in the green and red channels for the indicated genotypes (E: EGFP; C: mCherry). The number of analyzed glands (n) is indicated above the genotypes, and s.d. by whiskers. To facilitate comparisons, the same data already shown in S3 Fig is displayed again in case of the three left-most genotypes. Scale bars = 100 μm.
(PDF)

S5 Fig TEF self-association indicated by co-immunoprecipitation. S2R+ cells were transfected for transient co-expression of TEF-myc with either the N-terminal part of TEF fused to EGFP (TEF_N-E), the C-terminal part of TEF fused to EGFP (TEF_C-E) or nls-tetracycline-repressor fused to EGFP (TetR-E) for control as indicated. After extract preparation, antibodies against EGFP (anti-E) were used for immunoprecipitation. Input extracts and immunoprecipitated proteins were analyzed by immunoblotting with anti-E and anti-myc. A non-specific band recognized by anti-E is marked (*).
(PDF)

S6 Fig Localization of AHC proteins after ectopic expression during embryonic division cycle 14. (**A**) SUM (top), SUMT (middle) or TEF-EGFP (bottom) were expressed during embryonic division cycle 14 with maternally derived GAL4-VP16 from paternally inherited *UASt* transgenes. In case of SUM and SUMT, untagged AHC proteins were expressed, except for UNO, which was tagged with mCherry. Cells during the indicated cell cycle stages are displayed after fixation and DNA staining of gastrulating embryos. Centrosomes that bind TEF-EGFP during mitosis are indicated (arrowheads). (**B**) SUMT was expressed during embryonic division cycle 14 with maternally derived GAL4-VP16 from paternally inherited *UASt* transgenes coding for UNO-mCherry, TEF-EGFP and untagged SNM and MNM. A region from a gastrulating embryo with domains of cells at the indicated cell cycle stages is displayed after fixation and DNA staining. An anaphase figure within the domain of M14 cells is indicated (arrowheads). Scale bars = 5 μm (A) and 10 μm (B).
(PDF)

S7 Fig Effects of ectopic TEF-EGFP and SUM expression in wing imaginal discs. (**A**) The indicated AHC proteins were expressed in the posterior compartment of wing imaginal discs during 16 hours of incubation at 29°C before fixation and DNA staining. A region from the wing pouch is shown with the compartment boundary (dashed line) separating non-expressing control cells in the anterior compartment (left) from AHC protein-expressing cells in the posterior compartment (right). Normal anaphase and telophase figures are present in the anterior compartments (box 1), as well as in the posterior compartment (box 2) of discs that express either no AHC proteins (top) or TEF-EGFP (middle). In contrast, abnormal late mitotic figures were present in the posterior compartment (box 2) after ectopic expression of SUM (untagged SNM and MNM in combination with UNO-mCherry) (bottom). (**B**) SUM was expressed in imaginal wing discs as described above (A). Dissected wing imaginal discs were stained with a live DNA stain and analyzed by time-lapse imaging at 1 min intervals. A representative cell from the posterior SUM-expressing compartment is shown during exit from mitosis. Time (min) with t = 0 representing the last metaphase frame is indicated. Note that UNO-mCherry signals are occluded by the intense red fluorescent DNA stain. Scale bars = 10 μm (A) and 2 μm (C).
(PDF)

S1 Movie M I in *tef* mutant spermatocyte. Progression through M I as revealed by time-lapse imaging of a *tef* mutant spermatocyte expressing His2Av-mRFP, Cenp-A/Cid-EGFP and MNM-EGFP. A sequence of maximum intensity projections of the same cell as shown in Fig 1A–1D, after acquisition of z-stacks at 10-second intervals, is presented twice. The first time, with centromeres of chrX and chrY marked by comet tails, highlights the presence of a normal sex chromosome bivalent that displays correct bi-orientation in the equatorial plane during metaphase followed by regular separation during anaphase. The second time, comet tails marking the chr4 centromeres highlights premature bivalent separation followed by eventual missegregation of both chr4 univalents to the same spindle pole. Time (h:min:sec) is indicated.
(MP4)

S2 Movie Chromosomal mCherry-UNO-EGFP signals on autosomal and sex chromosome bivalent during early prometaphase I. The spermatocyte displayed in Fig 3E, as observed by time lapse imaging of mCherry-UNO-EGFP during early prometaphase I is shown. A large yellow circle surrounds the signal on the sex chromosome bivalent, and two smaller yellow circles the signals on autosomal bivalents. Time points with mCherry-UNO-EGFP signals sufficiently separated in space were used for quantification, followed by averaging intensities over these time points. Images were acquired without saturation of mCherry-UNO-EGFP signals. However, to reveal the weak autosomal signals, signal intensities were enhanced for production of the movie, resulting in saturation of the sex chromosome signals. Time (h:min:sec) is indicated.
(MP4)

S3 Movie M I in *bam*>*tef-EGFP* spermatocytes. Progression through M I was analyzed by time-lapse imaging of spermatocytes from *bam>tef-EGFP* males that also expressed *His2Av-mRFP*. Maximum intensity projections are displayed with TEF-EGFP in green and His2Av-mRFP in magenta. TEF-EGFP can no longer be detected during M I, which proceeds normally. Time (h:min:sec) is indicated.
(MP4)

S4 Movie M I in *bam*>SUM *(uno-mCherry)* spermatocytes. Progression through M I was analyzed by time-lapse imaging of spermatocytes from *bam>* SUM *(uno-mCherry)* males that also expressed *His2Av-GFP*. Maximum intensity projections are displayed with UNO-mCherry in green (with identical settings for image acquisition and display as those used for S5 Movie) and His2Av-mRFP in magenta. Time (h:min:sec) is indicated.
(MP4)

S5 Movie M I in *bam*>SUMT *(uno-mCherry)* spermatocytes. Progression through M I was analyzed by time-lapse imaging of spermatocytes from *bam>* SUMT *(uno-mCherry)* males that also expressed *His2Av-GFP*. Maximum intensity projections are displayed with UNO-mCherry in green (with identical settings for image acquisition and display as those used for S4 Movie) and His2Av-mRFP in magenta. Time (h:min:sec) is indicated.
(MP4)

S1 Table Summary of the effects of ectopic AHC protein expression. (XLSX)

S2 Table Description of the analyzed genotypes. (XLSX)

S3 Table Synthetic DNA fragments. (XLSX)

S4 Table Source data. (XLSX)
