# Supplementary figures and images for: Teflon promotes chromosomal recruitment of homolog conjunction proteins during Drosophila male meiosis
Source: PLoS Genet. 2022 Oct 17;18(10):e1010469. doi: 10.1371/journal.pgen.1010469 (PMC9612826; doi:10.1371/journal.pgen.1010469)

**A**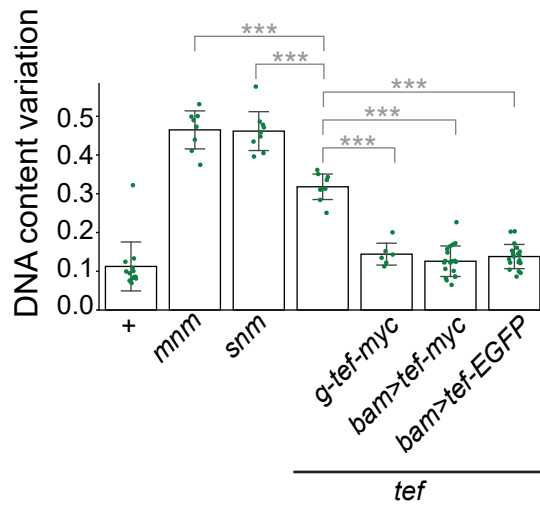**B**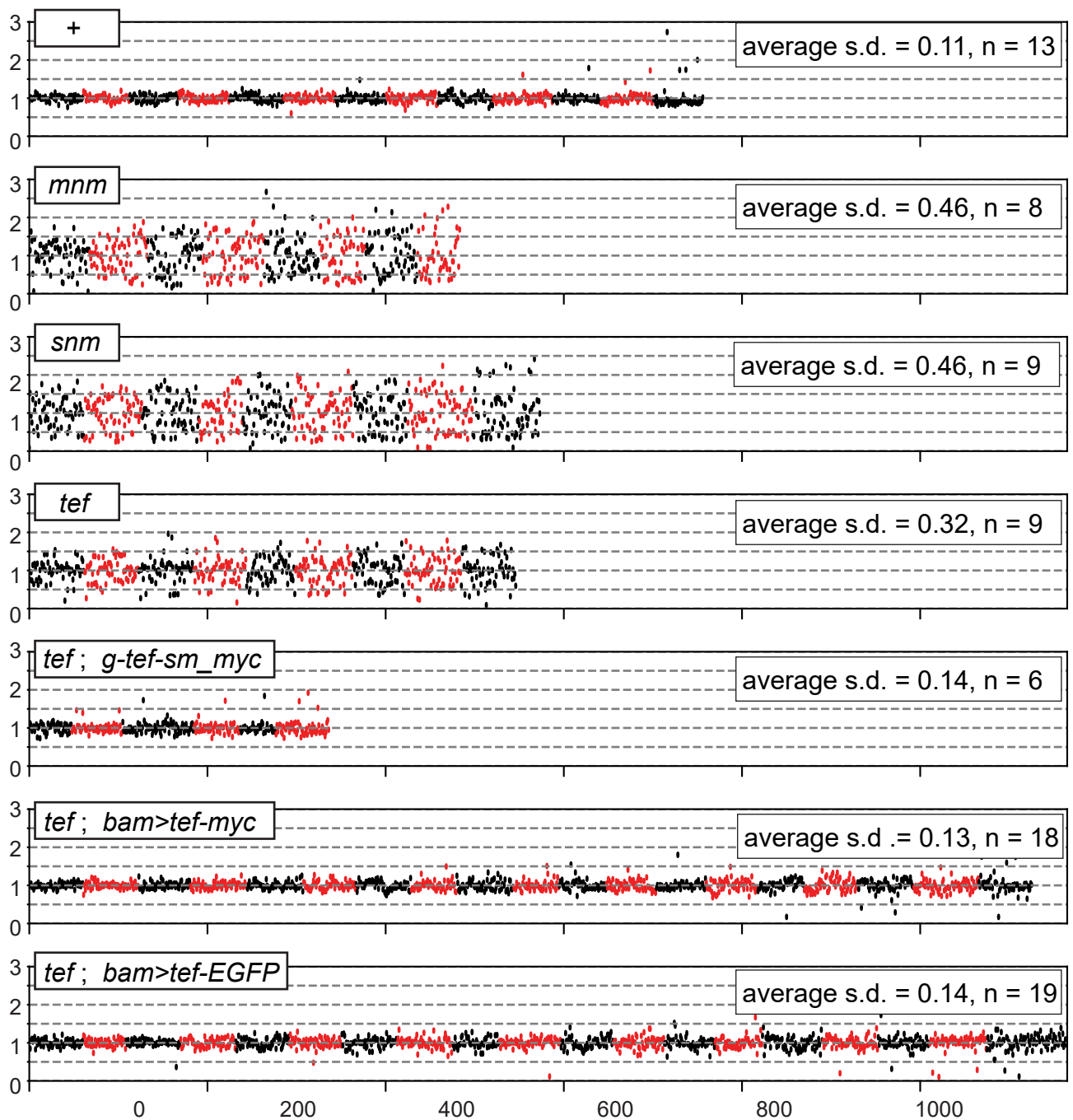

Supplement: S1 Fig — (A,B) DNA signal intensity was quantified for each early spermatid nucleus after imaging squash preparations of testes labeled with a DNA stain. The mean of all the individual nuclear DNA content values obtained for a given cyst was used for normalization of these values, followed by calculation of a normalized standard deviation (nsd). The analyzed genotypes were w (+), mnmz3-3298/mnmz3-5578 (mnm), snmz3-0317/snmz3-2138 (snm), tefz2-3455/tefz2-4169 (tef), tefz2-3455/tefz2-4169; g-tef-sm_myc III.1 (tef, g-tef-myc), tefz2-3455/tefz2-4169; bamP-GAL4-VP16/UASt-tef-myc III.1 (tef, bam> tef-myc), and tefz2-3455/tefz2-4169; bamP-GAL4-VP16/UASt-tef-EGFP III.2 (tef, bam> tef-EGFP). (A) Bar diagram summarizing DNA content variation. Each green dot represents the nsd of a given cyst. Bars indicate the nsd average across all analyzed cysts with whiskers displaying its standard deviation. DNA content variation in tef mutants is significantly different from that in mnm and snm mutants, and from that in tef mutants with the indicated tef transgenes (*** p < .001, t-test). (B) Plots displaying the normalized DNA content values for all the analyzed nuclei. Values from a given cyst are displayed in the same color, alternating between black and red for different cysts. The cysts analyzed for a given genotype are arranged with increasing nsd from left to right. The nsd average and the number of analyzed cysts are indicated in the upper right boxes. (PDF) [file pgen.1010469.s001.pdf]

**A**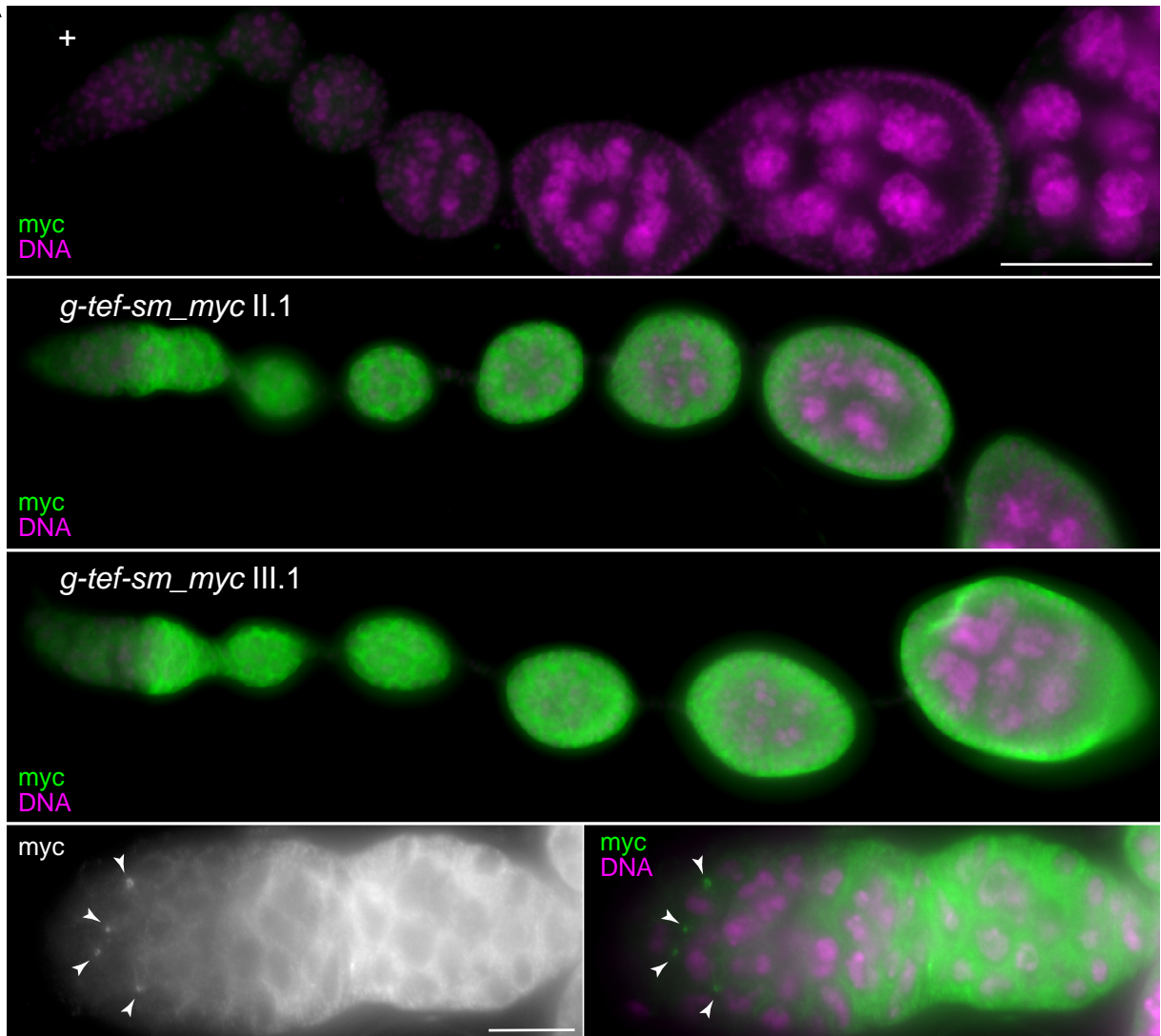**B**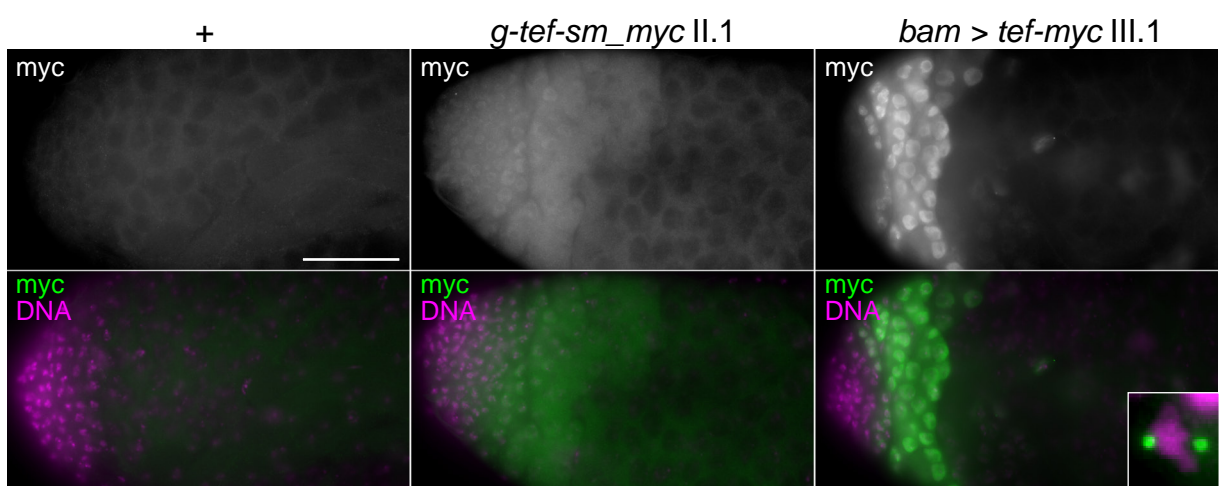**C**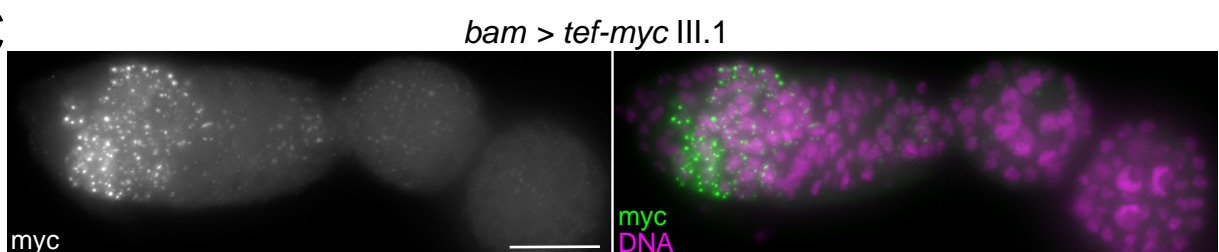

Supplement: S2 Fig — (A) Ovarioles from w1118 control (+) and g-tef-sm_myc transgenic females with either the insertion II.1 or III.1, as indicated, are shown after immunolabeling with anti-myc and a DNA stain. A high magnification of a germarium is shown in the bottom row with arrowheads indicating spindle poles in a mitotic oogonial cyst. (B) Whole-mount preparations of testes from w1118 control (+), g-tef-sm_myc II.1 and bam>tef-myc III.1 males were immunolabeled with anti-myc and a DNA stain. Identical exposure and display settings were used for the control and g-tef-sm_myc II.1 images, but an adjusted lower sensitivity was used in case of bam>tef-myc III.1 in order to avoid signal saturation in the green channel (anti-myc). A spermatogonial cell in metaphase with anti-myc signals on spindle poles is shown at high magnification (inset). In these whole-mount preparations, the specific anti-myc signals were overall less intense and less granular compared to those in flat testis preparations (Fig 2). (C) Germarium and early egg chambers from an ovariole of a bam>tef-myc III.1 female after immunolabeling with anti-myc and a DNA stain. Scale bars = 50 μm (A, top three rows), 10 μm (A, bottom row), 50 μm (B) and 20 μm (C). (PDF) [file pgen.1010469.s002.pdf]

**A**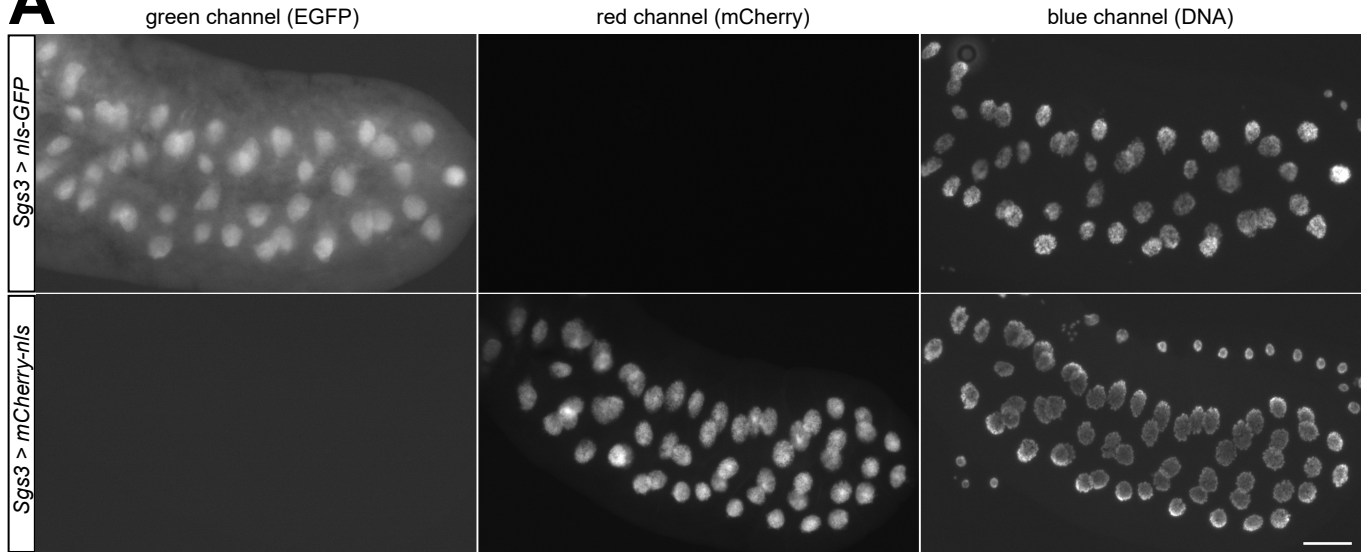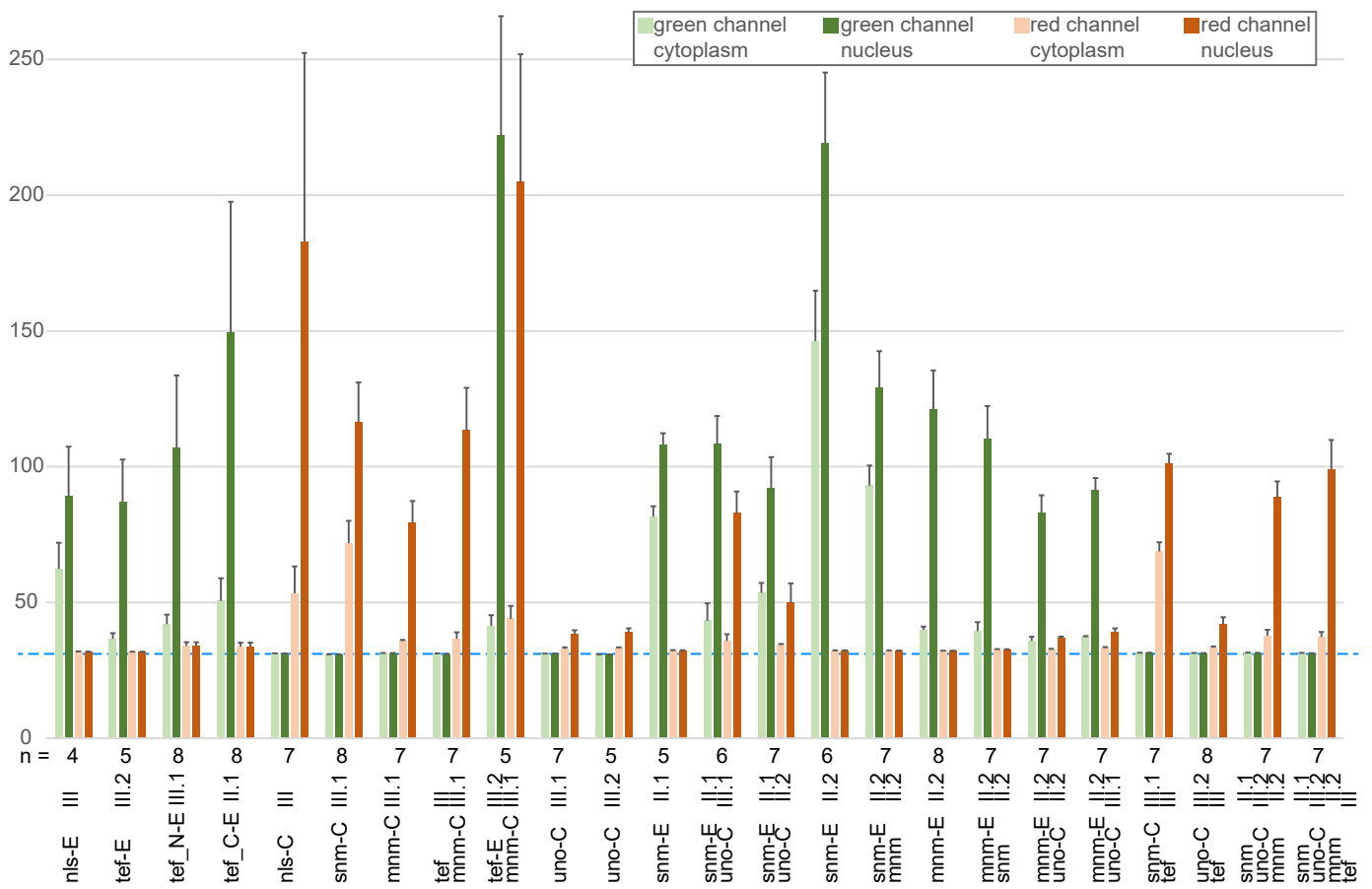**B**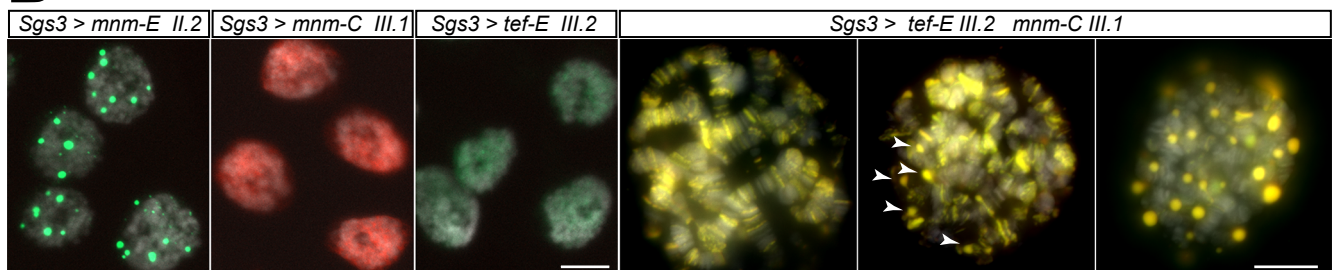

Supplement: S3 Fig — (A,B) Whole-mount preparations of larval salivary glands stained for DNA were used for comparison of the expression levels of various fluorescently tagged AHC proteins after expression with UASt transgenes and Sgs3-GAL4. (A) Representative images acquired using a 10x objective from the distal part of the gland with the basal cells from Sgs3>nls-GFP (top) and Sgs3>nls-mCherry (bottom) larvae. Identical exposure times were used for acquisition of a single equatorial focal plane from the green, red and blue channels for all the genotypes. Bar diagram displays mean pixel intensities (a.u.) detected in either the cytoplasm or in the nucleus in the green and red channels for the indicated genotypes (E: EGFP; C: mCherry). The number of analyzed glands (n) is indicated above the genotypes and s.d. by whiskers. The dashed blue line indicates background fluorescence intensity observed in the absence of expression of a fusion with EGFP or mCherry. (B) MNM-EGFP droplet formation. The three images on the left side display sub-regions of glands imaged as described above. The three images shown on the right side display images of nuclei acquired with a 100x objective (maximum intensity projections of four optical sections with 500 nm spacing). All images show a merge of the green, red and blue channels, with the DNA staining in the blue channel as grey values. Strong expression of MNM-EGFP results in the formation of intranuclear droplets. In contrast, such droplets are not detected after expression of MNM-mCherry or TEF-EGFP. The latter two fusion proteins are precisely co-localized after co-expression, shifting from chromosome-associated bands into intranuclear droplets (arrowheads in middle panel) with increasing expression levels. Scale bars = 100 μm (A), 50 μm (B, left side) and 10 μm (B, right side). (PDF) [file pgen.1010469.s003.pdf]

**Sgs3 > tef-EGFP**

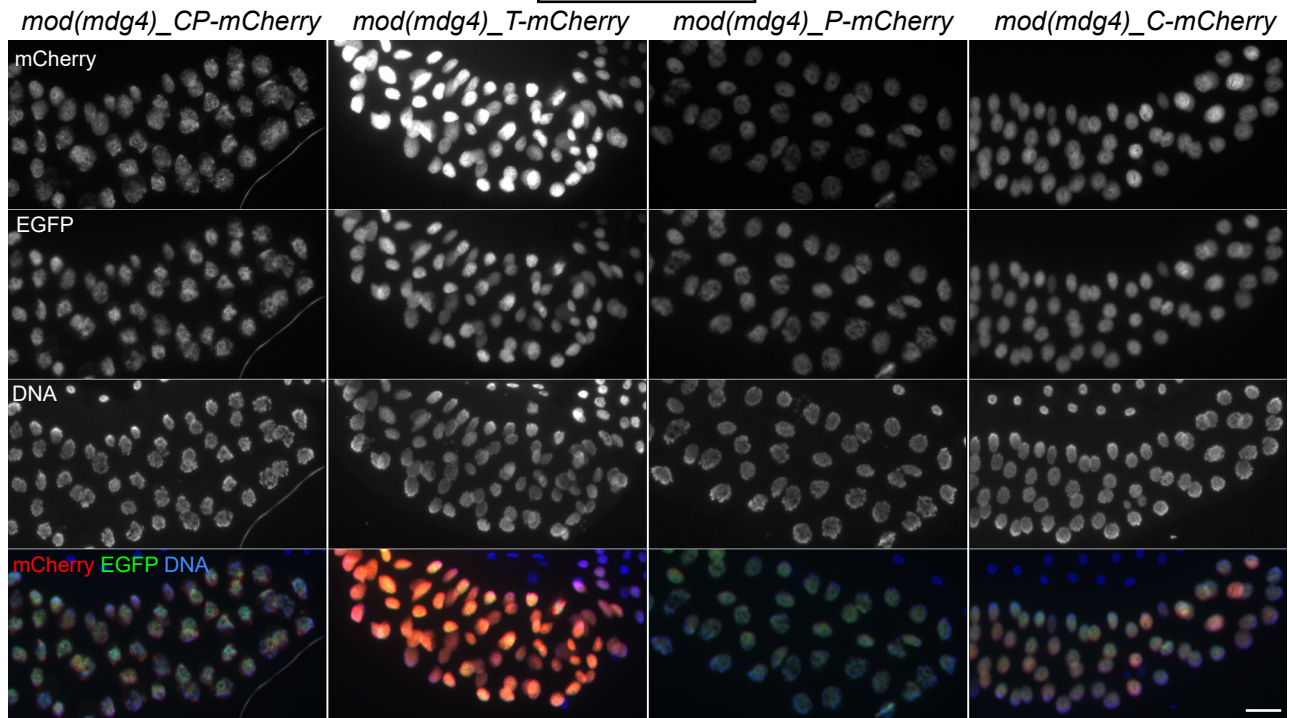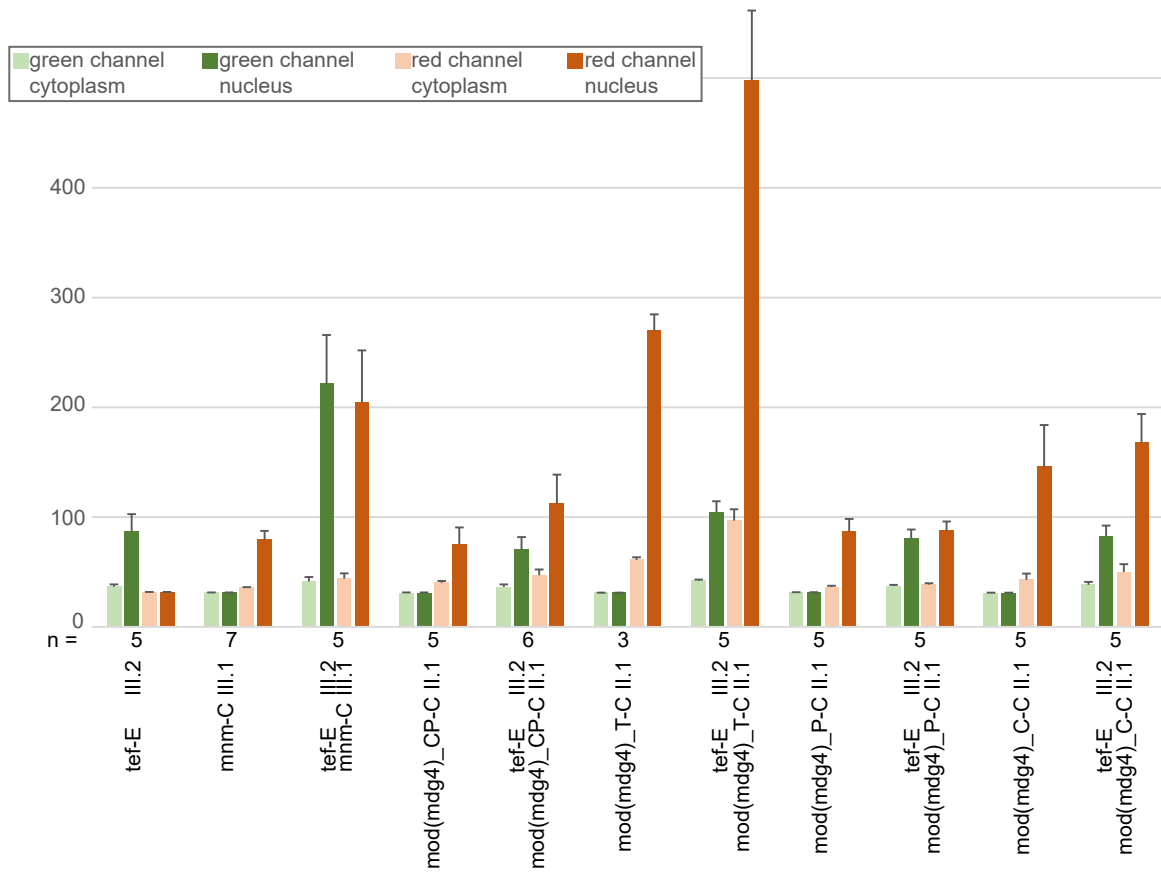

Supplement: S4 Fig — Sgs3-GAL4 and UASt transgenes coding for TEF-EGFP and the Mod(mdg4) isoforms CP, T, P and C tagged with mCherry were used for expression specifically in larval salivary glands. Whole-mount preparations were stained for DNA and imaged using identical exposure times. Representative single optical sections of the regions with the distal basal cells of the indicated genotypes are shown (top). Bar diagram displays mean pixel intensities (a.u.) detected in either the cytoplasm or the nucleus in the green and red channels for the indicated genotypes (E: EGFP; C: mCherry). The number of analyzed glands (n) is indicated above the genotypes, and s.d. by whiskers. To facilitate comparisons, the same data already shown in S3 Fig is displayed again in case of the three left-most genotypes. Scale bars = 100 μm. (PDF) [file pgen.1010469.s004.pdf]

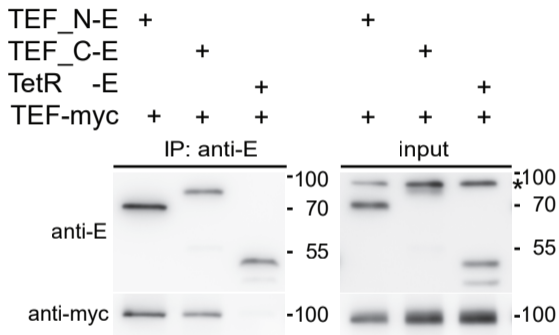

Supplement: S5 Fig — S2R+ cells were transfected for transient co-expression of TEF-myc with either the N-terminal part of TEF fused to EGFP (TEF_N-E), the C-terminal part of TEF fused to EGFP (TEF_C-E) or nls-tetracycline-repressor fused to EGFP (TetR-E) for control as indicated. After extract preparation, antibodies against EGFP (anti-E) were used for immunoprecipitation. Input extracts and immunoprecipitated proteins were analyzed by immunoblotting with anti-E and anti-myc. A non-specific band recognized by anti-E is marked (*). (PDF) [file pgen.1010469.s005.pdf]

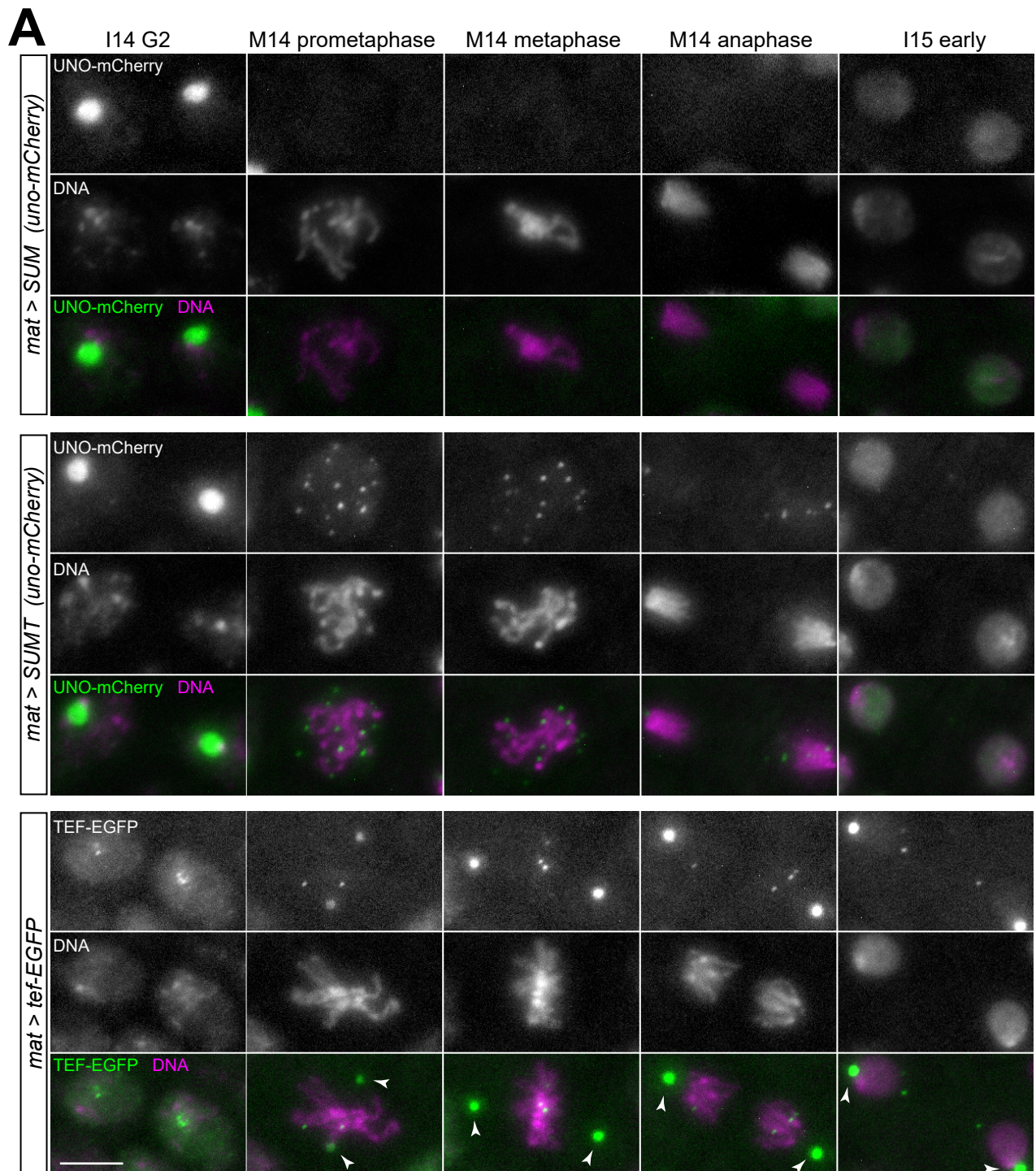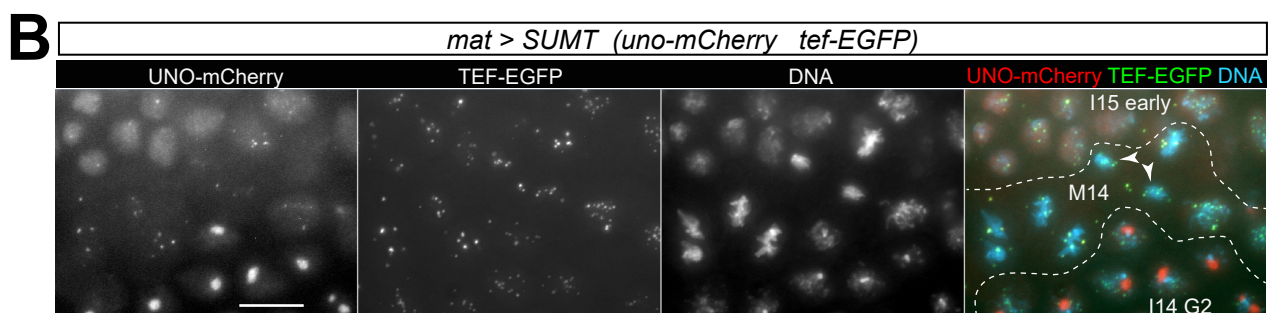

Supplement: S6 Fig — (A) SUM (top), SUMT (middle) or TEF-EGFP (bottom) were expressed during embryonic division cycle 14 with maternally derived GAL4-VP16 from paternally inherited UASt transgenes. In case of SUM and SUMT, untagged AHC proteins were expressed, except for UNO, which was tagged with mCherry. Cells during the indicated cell cycle stages are displayed after fixation and DNA staining of gastrulating embryos. Centrosomes that bind TEF-EGFP during mitosis are indicated (arrowheads). (B) SUMT was expressed during embryonic division cycle 14 with maternally derived GAL4-VP16 from paternally inherited UASt transgenes coding for UNO-mCherry, TEF-EGFP and untagged SNM and MNM. A region from a gastrulating embryo with domains of cells at the indicated cell cycle stages is displayed after fixation and DNA staining. An anaphase figure within the domain of M14 cells is indicated (arrowheads). Scale bars = 5 μm (A) and 10 μm (B). (PDF) [file pgen.1010469.s006.pdf]

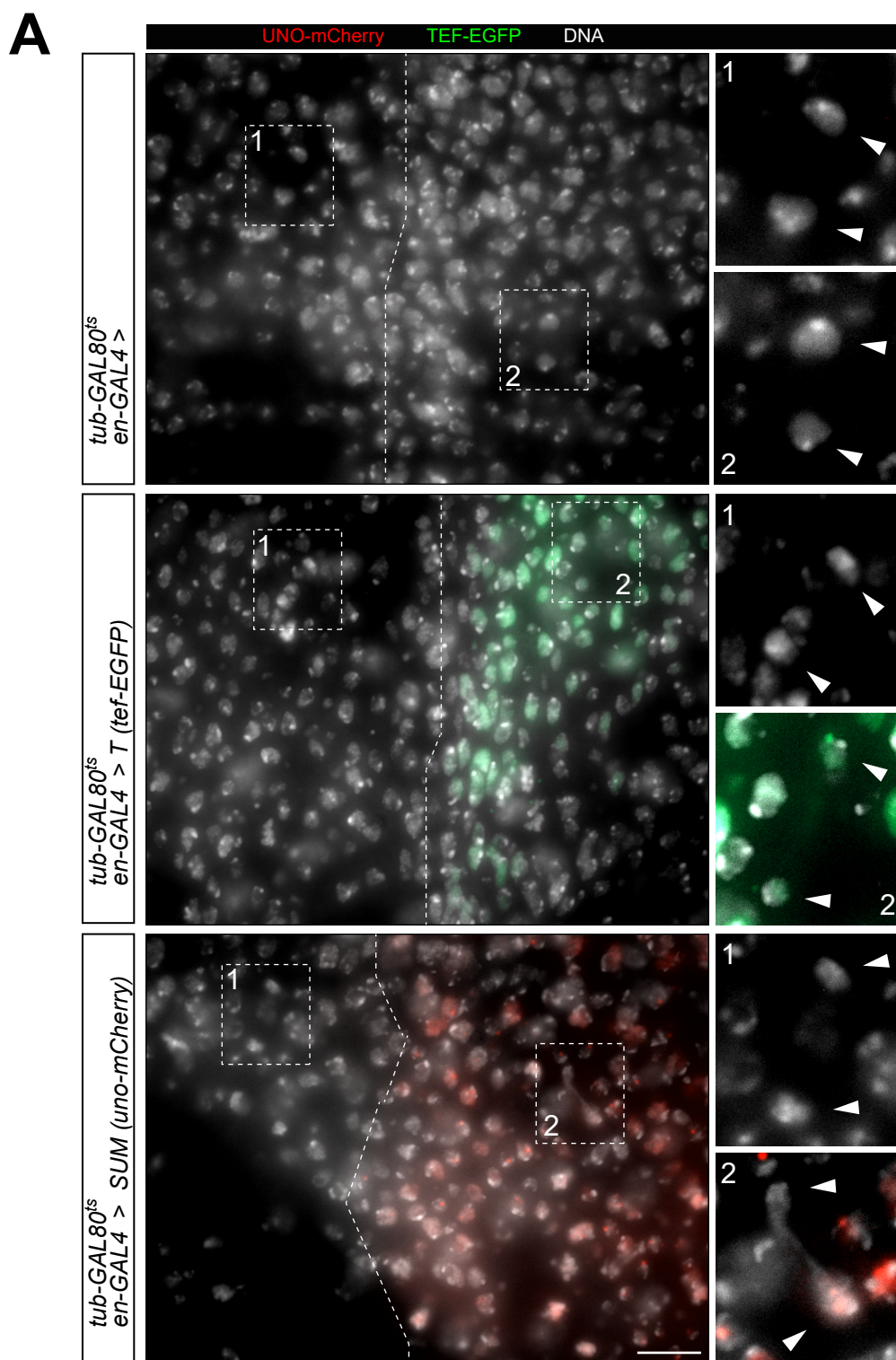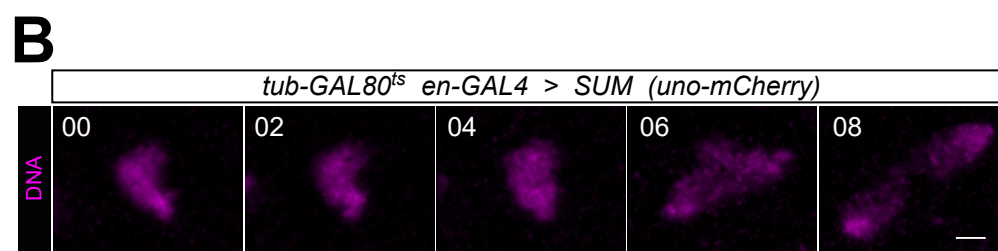

Supplement: S7 Fig — (A) The indicated AHC proteins were expressed in the posterior compartment of wing imaginal discs during 16 hours of incubation at 29°C before fixation and DNA staining. A region from the wing pouch is shown with the compartment boundary (dashed line) separating non-expressing control cells in the anterior compartment (left) from AHC protein-expressing cells in the posterior compartment (right). Normal anaphase and telophase figures are present in the anterior compartments (box 1), as well as in the posterior compartment (box 2) of discs that express either no AHC proteins (top) or TEF-EGFP (middle). In contrast, abnormal late mitotic figures were present in the posterior compartment (box 2) after ectopic expression of SUM (untagged SNM and MNM in combination with UNO-mCherry) (bottom). (B) SUM was expressed in imaginal wing discs as described above (A). Dissected wing imaginal discs were stained with a live DNA stain and analyzed by time-lapse imaging at 1 min intervals. A representative cell from the posterior SUM-expressing compartment is shown during exit from mitosis. Time (min) with t = 0 representing the last metaphase frame is indicated. Note that UNO-mCherry signals are occluded by the intense red fluorescent DNA stain. Scale bars = 10 μm (A) and 2 μm (C). (PDF) [file pgen.1010469.s007.pdf]
